# Multimodal Dynamics in Early Triadic Interactions at Home: A Longitudinal Analysis Throughout Infants' First Year of Life

**DOI:** 10.1111/infa.70136

**Published:** 2026-11-13

**Authors:** Ana Mendoza‐García, Ana Moreno‐Núñez, Andrés Aparicio

**Affiliations:** ^1^ Departamento Interfacultativo de Psicología Evolutiva y de la Educación. Facultad de Psicología Universidad Autónoma de Madrid Madrid Spain; ^2^ Facultad de Ciencias de la Rehabilitación y Calidad de Vida Universidad San Sebastián Santiago Chile; ^3^ Millennium Institute of Care Research (MICARE) Santiago Chile

**Keywords:** communicative development, infant‐caregiver interaction, multimodal communication, object use, triadic interaction

## Abstract

Early communicative development during the first year of life unfolds through rich, multimodal exchanges in which infants and caregivers coordinate multiple resources—such as gestures, object use, vocalizations, and touch—into dynamic patterns that shape early language trajectories. However, less is known about how these behaviors evolve for both partners in triadic contexts, where objects anchor joint engagement, and how they are associated with language development. This longitudinal study followed nine infant‐caregiver dyads with monthly behavioral observations from 2 to 12 months during naturalistic free‐play interactions at home. We frame‐by‐frame coded infant behaviors (spontaneous movements, rhythmic movements, gestures, vocalizations) and adult behaviors (distal object‐use demonstrations of varying complexity, rhythmic and non‐rhythmic touch). Analyses examined age‐related change, multimodal combinations, and their associations with MacArthur‐Bates CDI comprehension and production trajectories, assessed at 9, 12, and 16 months. Infants showed a decline in spontaneous movements across the first year, while vocalizations were more often produced in isolation than alongside manual gestures. Multimodal combinations involving rhythmic movements with objects increased over time, whereas gesture‐vocalization combinations remained rare and did not show significant age‐related increases. In adults, simple object‐use demonstrations decreased, whereas more complex demonstrations and non‐rhythmic touch became relatively more prominent, although both proximal and distal interaction strategies decreased overall with age. Certain adult behaviors—particularly symbolic object‐use demonstrations—were associated with infants' language‐growth trajectories. Several infant behavioral trajectories were also associated with language growth, with these associations varying as a function of estimated language level at 12.3 months. These findings reveal intertwined infant‐caregiver developmental trajectories and underscore that early communication in triadic contexts relies on coordinated motor, vocal, and material channels sustained by shared engagement with objects.

## 1 Introduction

From the earliest months of life, early communication takes shape within meaningful, embodied, and contextually situated interactions among infants, their caregivers, and objects in their everyday environment (Carpendale et al. 2021; Rossmanith et al. 2014). These early exchanges have long been described as foundational to intersubjectivity and shared meaning‐making (Trevarthen and Aitken 2001), as infants progressively participate in structured social *formats* and coordinated routines that organize attention and action (Bruner 1981, 1983). Within these exchanges, interaction is organized through dyadic (adult–infant) and triadic (adult–object–infant) configurations that structure participation from early in the first year. These triadic interactions are characterized by multimodal communicative exchanges organized into joint sequences of attention and action mediated by materiality, through which adults and infants become jointly engaged with objects over time (De Barbaro et al. 2016; Mendoza‐García and Moreno‐Núñez 2023). Objects not only mediate interaction but also create opportunities for action, guide temporal and social coordination, and reorganize behavior around shared usage patterns, as object use is socially demonstrated and appropriated within interaction (Azagra et al. 2026), thereby fostering the sociocultural construction of meaning and infants' participation in social life (Solovieva and Quintanar 2021; Vygotsky 1996). In this sense, material engagement contributes to the stabilization of shared reference and to the progressive differentiation of communicative participation across development.

The timing of the emergence of triadic participation has been the subject of considerable debate. Classical perspectives place its onset between 9 and 12 months, referring specifically to the emergence of infants' intentional triadic communication, coinciding with the emergence of deictic gestures (Tomasello 2013). Within this framework, triadic participation is identified with the emergence of explicit intentional communication and coordinated attention toward objects. In contrast, dynamic systems and post‐cognitivist approaches propose that while intentional communication becomes more clearly observable from this period onward, it is grounded in a prior and gradual developmental process shaped by adult scaffolding and materiality (Fogel and Thelen 1987; Fogel 1993; Reddy 2010; Thelen and Smith 1994). From these perspectives, exchanges are bidirectional, and objects serve as communicative anchors and organizational structures from the first months of life (Moreno‐Núñez et al. 2017), within a process of joint meaning‐making. Triadic participation can thus be understood as progressively elaborated within socially structured and materially embedded interactional contexts.

In the present study, we distinguish between three related but analytically distinct levels within early interaction. First, triadic interaction refers to the interactional configuration involving infant, caregiver, and object. Second, multimodal coordination refers to the process through which different behavioral modalities (e.g., movement, gesture, vocalization) temporally co‐occur and become organized within interaction. Finally, communication refers to the functional dimension of these exchanges, when coordinated behaviors contribute to influencing or orienting a partner's attention or action. The present analyses focus primarily on observable patterns of multimodal coordination within triadic interaction, and do not directly assess communicative intentionality.

### 1.1 Infant Motor and Communicative Development: The Role of Multimodal Coordination

Well before producing their first words, typically around 10–12 months of age, infants engage in intersubjective experiences, understood as early forms of shared engagement with others, such as affective exchanges, turn‐taking interactions, and shared participation in everyday routines. These early exchanges are grounded in embodied action and sensorimotor coordination, which progressively expand infants' possibilities for social participation (Iverson and Fagan 2004; Thelen and Smith 1994). These experiences stimulate their communicative intentionality, defined as the emerging capacity to act in ways oriented toward influencing others, for example when infants point to request an out‐of‐reach object from an adult or show an object of interest to share attention with them (Papoušek 2007; Stern 2018; Trevarthen 1999).

Within this developmental process, changes in motor abilities play a central role in reorganizing infants' engagement with their social environment. Developmental research has highlighted close links between advances in motor abilities and emerging communicative behaviors during the first year of life (Iverson and Fagan 2004). For example, empirical studies have shown that the emergence of new postural and locomotor abilities can transform infants' opportunities to interact with objects and social partners, with cascading effects on communicative and linguistic development (Iverson 2010). Longitudinal research has further documented associations between the onset of independent sitting and later receptive vocabulary growth (Libertus and Violi 2016), while infants who begin walking earlier tend to show larger receptive and productive vocabularies than crawling peers, even when controlling for age (He et al. 2015). In particular, changes in postural control, reaching, and manual exploration alter infants' access to objects and partners, reshaping the contexts in which interaction unfolds.

Early spontaneous movements described in the framework of *General Movements* (Prechtl 1990, 1997) and rhythmic motor patterns documented in dynamic systems research (Thelen 1979, 1981) reflect organized forms of motor activity that precede later goal‐directed actions. These early motor patterns have been proposed to constitute part of the developmental groundwork from which more differentiated forms of action and communicative behavior may emerge, as initially undifferentiated movements become progressively reorganized into more goal‐directed and socially oriented actions (Azagra et al. 2026).

As motor capacities expand, infants participate more actively in shared routines. Language learning is not limited to exposure to words but is grounded in active participation in shared routines where bodily activity, material exploration, and social coordination converge (e.g., during play, caregiving activities, etc.) (Iverson and Fagan 2004; Murillo et al. 2018; Vigliocco et al. 2014). Communication is thus understood as a fundamentally multimodal process, co‐constructed through the dynamic integration of semiotic resources and material engagement.

Far from being passive recipients, infants co‐construct the flow of interaction, gradually integrating into it through resources such as gaze (Northrup and Iverson 2020), vocalizations (Albert et al. 2023), gestures (Murillo et al. 2018), and body movements (Moreno‐Núñez et al. 2021; Murillo et al. 2021). This active participation becomes particularly salient when multiple modalities are combined simultaneously: the likelihood of receiving a contingent response increases substantially when elements such as gaze, vocalization, and movement overlap (Moreno‐Núñez et al. 2021). Across the first year, the coordination of these resources becomes increasingly organized, reflecting a progressive differentiation of infants' multimodal participation in interaction (Azagra et al. 2026). Multimodal synchrony—defined as the temporal overlap between modalities—has been associated with better word comprehension at 12 months and greater word production at 18 months (Murillo et al. 2018).

### 1.2 Adult Scaffolding in Object‐Centered Interactions

In object‐centered exchanges, caregivers actively structure infants' participation through a coordinated repertoire of multimodal resources. Their verbal contributions—such as comments, questions, and contingent responses adapted to the moment and to the child's characteristics (Dimitrova et al. 2015)—are often embedded within the prosodic and lexico‐syntactic patterns typical of child‐directed speech (Cameron‐Faulkner et al. 2003), which highlight relevant information and support early lexical acquisition (Albert et al. 2023). These verbal strategies are typically temporally aligned with embodied and object‐related actions that shape infants' attention in real time.

From early in the first year, caregivers frequently rely on proximal strategies, physically organizing infants' engagement by touching, repositioning, or manipulating objects within shared space. Such embodied scaffolding reduces attentional demands and supports coordinated exploration (Gattis et al. 2020; Moreno‐Núñez et al. 2015). Touch also contributes to regulating attention and sustaining engagement, as physical contact can stabilize arousal, reinforce affective attunement, and anchor infants' attention within the shared activity (Fini et al. 2023; Murillo et al. 2025; Serra et al. 2020).

Across development, parental guidance increasingly incorporates distal strategies that direct infants' attention without physical contact. These include ostensive cues, gaze direction, and deictic gestures such as pointing (Iverson and Goldin‐Meadow 2005; Moreno‐Núñez et al. 2017). Developmental evidence indicates that infants progressively become able to interpret and integrate such distal signals—especially when multiple cues converge—toward the end of the first year (Deák et al. 2000; Sun and Yoshida 2022).

In parallel, a growing body of work has examined how parent–infant coordination around objects supports infants' visual attention and engagement. Caregivers have been shown to scaffold infants' access to objects by structuring clear views and coordinating their actions with the infant's focus of attention, thereby supporting sustained attention and object exploration (Burling and Yoshida 2019; Deák et al. 2014; Yu and Smith 2016).

Research on early interaction has highlighted the importance of both verbal and nonverbal forms of parental responsiveness for promoting early communicative and language development. Within this body of work, manual interactions with objects have been shown to facilitate word–object mapping, particularly during the preverbal stage (Gogate et al. 2000), supporting infants' ability to establish associations between words and the objects they refer to. Moreover, combining speech with object manipulation and touch has been linked to longer infant gaze toward the object (Suárez‐Rivera et al. 2019), indicating that coordinated speech–action patterns contribute to sustaining attention during episodes of joint engagement.

Together, this body of research shows that caregivers organize object‐centered interaction through developmentally shifting structuring strategies, including proximal embodied support and increasingly distal attentional guidance shaped by rhythmic and object‐related actions. Recent findings further indicate that distal demonstrations of object use may incorporate rhythmic structuring that adapts to infants' developmental stage between 6 and 12 months (Mendoza‐García et al. 2026), highlighting the developmental sensitivity of adult guidance within the first year of life.

Despite growing interest in early communicative development, longitudinal evidence describing how certain motor and communicative behaviors unfold across the first year of life remains limited. In particular, relatively few longitudinal studies have examined how early motor activity and emerging communicative behaviors develop within everyday interactional contexts during the first year of life. As a result, important questions remain unclear about how early motor and communicative behaviors become organized within early social interactions.

A previous study using this same sample (Azagra et al. 2026) documented longitudinal changes in infants' spontaneous movements, rhythmic movements, gestures, and vocalizations across the first year of life, as well as their associations with lexical development. The previous study documented a developmental sequence in which changes in spontaneous and rhythmic movements precede increases in intentional gestures over the first year of life. These findings provide initial longitudinal evidence linking early motor activity with the emergence of later communicative behaviors.

Building on these findings, the present study situates infants' motor and communicative behaviors within object‐centered interactions with caregivers. The analysis focuses on infants' early vocal–motor coordination before 12 months and considers aspects of adult scaffolding during these exchanges, particularly nonverbal behaviors such as touch and object‐related actions, while examining how these early developmental patterns are associated with vocabulary growth up to 16 months.

In the present study, sociomaterial engagement is approached both as a theoretical perspective and as an empirical dimension of analysis. Theoretically, it highlights the constitutive role of objects in early interaction and in the organization of infants' communicative activity. Empirically, this perspective is reflected in the coding scheme through the systematic distinction between behaviors occurring with and without object involvement, allowing us to examine how multimodal coordination unfolds within triadic interactional contexts.

Importantly, although the present study uses a dataset that has been analyzed in related work, it differs substantially in both scope and analytical approach. Previous analyses focused exclusively on infants' motor and communicative behaviors using a descriptive, coarse‐grained temporal framework. In contrast, the present study incorporates (a) object‐related versus non‐object‐related behaviors, (b) adult nonverbal behaviors, and (c) a fine‐grained longitudinal modeling approach, enabling the examination of developmental trajectories and cross‐domain associations that were not addressed in prior work.

## 2 Objectives and Hypotheses

Based on this framework, the study addresses the following objectives:

1. To characterize age‐related developmental patterns in the rates and duration of selected infant and adult behaviors across the first year of life, with categories differentiated by object involvement.
2. To examine the developmental organization of infants' interactive behaviors, including spontaneous movements, rhythmic movements, and gestures, in relation to their multimodal co‐occurrence with vocalizations over time.
3. To examine associations between changes in interactive behaviors and infants' communicative and linguistic development, as assessed at 9, 12, and 16 months.

Based on previous research on early multimodal communication and caregiver responsiveness, we formulate the following hypotheses addressing developmental change across the first year and associations with language development trajectories:

H 1a We hypothesize that infants' interactive behaviors show age‐related changes across the first year of life, with spontaneous movements predominating in earlier months, followed by rhythmic movements, and gestures becoming more frequent later on, reflecting increasing motor organization and social coordination.

H 1b We hypothesize that parental nonverbal responsiveness evolves with age, with proximal strategies (e.g., bodily touch) predominating in the early months and more distal strategies (e.g., demonstrations of object use) becoming more frequent later on, in line with infants' increasing motor and attentional capacities.

H 2 We expect that the rates of multimodal combinations in infants' behaviors (e.g., movements or gestures accompanied by vocalizations) increase with age, reflecting the gradual integration of motor and vocal systems during the first year.

H 3 Finally, we hypothesize that individual differences in infants' interactive behaviors (movements, gestures, vocalizations, and their multimodal combinations) will be associated with individual trajectories of language comprehension and production across 9, 12, and 16 months.

## 3 Method

### 3.1 Participants

The sample consisted of nine adult‐infant dyads, including seven mothers and two fathers interacting with their infants (four girls). All participants came from Spanish middle‐class families with a university‐level education. The mean age of adults at the beginning of the study was 35.47 years (SD = 2.92). All infants were born at term and held Spanish nationality. Most dyads (*n* = 7) resided in the metropolitan area of Madrid and were monolingual Spanish‐speaking. Two families resided in other regions outside Madrid. In these households, infants were also exposed to additional languages (Galician, Catalan, or French) through parental input at home.

Participants were recruited through purposive sampling within the research team's professional and community networks based in Madrid (e.g., early childhood centers and parent groups), ensuring families' availability for repeated longitudinal participation. Infants were approximately 2 months (*M* = 2.08 months, SD = 0.22) at the beginning of the study. Although the sample size was small, this choice is justified by the microanalytic nature of the design, which focused on documenting in detail the complexity, variability, and evolution of triadic interactions in natural contexts during the first year of life. Small‐sample idiographic or microgenetic designs are common in research on early communicative development, as they enable systematic analysis of high‐frequency multimodal behaviors during critical developmental periods (Bakeman and Adamson 1984; Fogel 1993; Rogoff 2003). Comparable longitudinal multimodal studies using intensive microanalytic coding procedures have likewise relied on small samples due to the depth and temporal resolution of the analyses involved (e.g., Alessandroni et al., 2020; Moreno‐Núñez et al. 2015; Rossmanith et al. 2014). In addition, the present design involved repeated observations across 11 sessions per dyad, generating a substantial corpus of fine‐grained multimodal data. Data collection continued until all 11 planned sessions per dyad were completed, following a predefined protocol and with no attrition. Table 1 summarizes the main sociodemographic characteristics of the sample.

**TABLE 1:** Sociodemographic information of the study participants.

Variables	Adults	Infants
*N* total	9	9
*N* females	7	4
Age at start of study	35.47 years (SD = 2.92)	2.08 months (SD = 0.22)
Age at end of study	36.30 years (SD = 2.92)	12.15 months (SD = 0.08)
% Attendance at childcare center	NA	66.66%
Age at childcare entry	NA	9.33 months (SD = 2.80)
% Children exposed to other languages^a^	NA	22.22%

^a^ Percentage of children whose parents reported exposure to at least one additional spoken language besides Spanish through interactions with their primary caregivers or other regular caregivers (e.g., parents, grandparents, or educators), regardless of the amount of exposure. Media exposure (e.g., television) was not considered.

All procedures were approved by the Research Ethics Committee of Universidad Autónoma de Madrid, and informed consent was obtained from all families in accordance with the ethical standards outlined in the Declaration of Helsinki.

### 3.2 Materials

For the observations, each family was provided with a small maraca to introduce a common object across sessions, following previous studies (Alessandroni et al. 2020; Mendoza‐García et al. 2026; Moreno‐Núñez et al. 2015, 2017, 2020). This rhythmic‐sonorous object, easy to grasp and manipulate, provided a minimal degree of standardization while preserving the naturalistic character of the interactions. Because recordings took place in families' everyday environments, interactions were not restricted to this object and infants and caregivers frequently incorporated other available household objects during free‐play episodes, particularly once infants began to move independently. The repeated availability of the maraca across sessions allowed infants to interact with a familiar artifact while still engaging with a changing set of other objects present in the home environment. Analyses were not object‐specific but focused on the presence versus absence of object involvement. The coding scheme therefore distinguished between behaviors occurring with objects and those occurring without object involvement, regardless of the specific object used.

Sessions were recorded using a JVC Everio GZ‐HM310 camera mounted on a tripod and placed at a side angle to the participants, ensuring that the framing clearly captured joint actions. No external audio recording devices were used.

### 3.3 Procedure

Each family was visited once per month when infants were between 2 and 12 months of age, resulting in a total of 99 observations (11 per dyad). Interactions took place in a free‐play setting involving the adult, the infant, and objects, without specific instructions on how to use the objects, in order to preserve the ecological validity of the interaction. The researcher remained silent and refrained from intervening during the recordings. Sessions were planned to last 10 minutes. Overall session duration averaged 10.37 minutes (SD = 0.93), with a range from 8.20 to 15.52 minutes. Only eight of the 99 recorded sessions lasted less than 10 minutes. These sessions were retained for analysis because they exceeded 8 minutes and provided sufficient material for reliable coding. If the infant showed discomfort before this time and the adult's strategies failed to reestablish the interaction, the session could be ended. In some cases, families chose to prolong the interaction beyond 10 minutes. However, for analysis purposes, only the first 10 minutes were coded to ensure a standardized observation window across participants. Frequency measures were expressed as rates per minute to account for minor variability in coded session length, and duration‐based measures were calculated proportionally relative to the total duration of each video to ensure comparability across sessions.

Following the declaration of the state of emergency in Spain in March 2020, and the restrictions imposed due to the COVID‐19 pandemic, the protocol was adapted so that families recorded the interactions in their homes using their own devices. The research team provided detailed instructions and online supervision, reviewing each submitted video to ensure that it was suitable for the study objectives. Families received specific guidelines regarding camera placement, framing, lighting conditions, and audio quality, with the aim of maintaining recording conditions comparable to those of the in‐person sessions. Each video was systematically reviewed by the research team to verify that both members of the dyad and their joint actions were clearly visible and that audio and lighting conditions were adequate for coding.

All recordings were conducted in the families' homes under naturalistic conditions, typically in common household spaces such as the living room or, in some cases, the child's play area. When researchers were present during the recordings, they remained silent and physically distant from the interaction area in order to preserve the naturalistic quality of the interactions. Across the dataset, researchers were present in 23 of the 99 recorded sessions (23.23%), whereas 76 sessions (76.77%) were conducted without a researcher present. However, all videos were reviewed to ensure comparable framing, visibility of actions, and audio quality.

Sessions were scheduled approximately once per month between 2 and 12 months of age. Families were contacted during the week in which the infant reached the target age, and the specific recording day was arranged within that same week according to family availability, resulting in intervals that generally approximated 1 month with only minor variations of a few days.

Information on infants' communicative and linguistic development was also collected using the Spanish adaptation of the MacArthur‐Bates Communicative Development Inventories (CDI; López Ornat et al. 2005). Parents completed Inventory I (8–15 months) at 9 and 12 months, and Inventory II (16–30 months) at 16 months. For this study, and following previous evidence (Murillo et al. 2018), only the word Production and Comprehension indexes—present in both CDI versions—were analyzed.

### 3.4 Data Coding

Recordings were coded using ELAN software (Version 6.7; Lausberg and Sloetjes 2009) following a category system based on previous work (Alessandroni et al. 2020; Azagra et al. 2026; Moreno‐Núñez et al. 2017, 2020; Moreno‐Núñez and Alessandroni 2021) and adapted for a frame‐by‐frame microanalytic analysis. The onset, duration, and type of each behavior were annotated, allowing identification of both simultaneous co‐occurrences and temporal sequences across modalities.

Infant behaviors analyzed included: (1) spontaneous movements, with and without objects; (2) rhythmic movements, with and without objects; (3) gestures, with and without objects; and (4) vocalizations, including babbling, isolated vocal sounds, and proto‐words (excluding crying, laughter, and vegetative sounds). Adult nonverbal behaviors included: (1) distal demonstrations of object use—defined as actions performed to show the infant how to manipulate the object, without direct physical contact with the baby, and classified into four mutually exclusive levels of complexity; and (2) bodily touches—classified as rhythmic (i.e., repetitive) or non‐rhythmic (i.e., single or irregular), including postural adjustments or caresses, with or without objects.

For multimodality analysis, combinations of infant behaviors were identified as temporal co‐occurrences between modalities, computed post hoc based on the overlap between independently annotated motor behaviors and vocalizations in ELAN. Any temporal co‐occurrence between behaviors was considered a multimodal event. The present study focuses on observable patterns of multimodal coordination and does not directly assess communicative intentionality. Three variables were created: (1) type of behavior (spontaneous movement, rhythmic movement, or gesture), (2) presence or absence of vocalization, and (3) presence or absence of object use. Table 2 summarizes these categories, including operational definitions and coding criteria.

**TABLE 2:** Behavioral categories used in this study.

Participant	Category	Description
Infant	Spontaneous movements	Non‐repetitive motor actions performed with the arms and/or hands, with or without objects. They lack clear intentional control and do not display a discernible rhythmic pattern. Includes gentle swaying or twisting of the body, in line with the general movements described by Prechtl and Hopkins (1986), such as *writhing* and *fidgety movements*. Coded when lasting at least 1 second and not interrupted by brief pauses (≤ 2 seconds).
Infant	Rhythmic movements	Repetitive motor behaviors with a regular temporal pattern, with or without objects. Includes actions such as rhythmically banging a surface or an object. Considered part of the typical *rhythmic stereotypies* of early sensorimotor development (Thelen 1981). The sequence is considered interrupted if there is at least a 2‐s pause or a qualitative change in the action.
Infant	Gesture	Manual or bodily actions with clear communicative intent. Includes pointing, showing, offering, clapping, or pretending, whether performed with or without objects. Gestures were coded as communicative when they showed clear orientation toward the caregiver (e.g., gaze directed to the partner, arm extension toward the adult, or persistence contingent on the partner's response).
Infant	Vocalizations	Vocal productions such as babbling, proto‐words, or emerging words. Crying, laughter, and vegetative sounds are excluded.
Adult	Distal demonstrations of object use	Actions in which the adult manipulates the maraca in front of the infant, without direct physical contact with the infant, in order to demonstrate how the object can be used. These demonstrations were coded into four mutually exclusive levels of increasing complexity: Level 1: Non‐rhythmic or unconventional handling (e.g., shaking the maraca upside down or spinning it in the air like a bell). Level 2: Striking the maraca against another surface (e.g., hand or floor). Level 3: Conventional maraca use, holding the handle to produce a rhythmic sound. Level 4: Symbolic uses representing something different from its typical function (e.g., pretending the maraca is a microphone or a bird flying toward the infant).
Adult	Rhythmic touch	Repetitive touches applied by the adult to the infant's body with a perceptible cadence. May or may not involve an object.
Adult	Non‐rhythmic touch	Single or irregular touches applied by the adult to the infant's body, without a repetitive pattern. Includes actions such as postural adjustments, caresses, or brief touches, with or without objects.

To ensure consistency in the application of coding criteria, interobserver reliability was assessed using partial samples of recorded sessions. A total of 22 out of 99 videos (22.22% of the total sample) were randomly selected from the full dataset and independently coded by a second trained observer for all behavioral categories. This proportion is consistent with commonly reported standards in observational studies of early communication. In all cases, the number of videos analyzed exceeded 10% of the total, consistent with recommendations for observational studies (Bakeman and Quera 2011), and falls within the range commonly reported in similar studies (e.g., 17%–20% in Carpenter et al. 1998, 25% in Lee et al., 2018).

For infants' motor behaviors, inter‐rater reliability was high. Cohen's Kappa coefficients were *κ* = 0.774 (94.2% agreement) for spontaneous movements and *κ* = 0.856 (97.8% agreement) for rhythmic movements. For gestures, reliability was *κ* = 0.976 (99.8% agreement).

Vocalizations were coded using a binary presence/absence criterion. Inter‐rater reliability for vocalizations was *κ* = 0.839 (97.4% agreement), indicating substantial agreement.

For adults' nonverbal behaviors, reliability was also high. Cohen's Kappa coefficients were *κ* = 0.819 (97.6% agreement) for rhythmic touches, *κ* = 0.836 (93.3% agreement) for non‐rhythmic touches, and *κ* = 0.703 (89.9% agreement) for distal demonstrations of object use.

### 3.5 Data Analysis

We implemented two complementary analysis plans (see Table 3 for an overview of the analytic strategy). In the first plan, we examined developmental changes in the rates and duration of communicative behaviors across the first year of life (objectives 1 and 2), by preparing separate datasets for adult, infant and multimodal behaviors. Age was mean‐centered within each dataset. For each dataset and dependent variable (duration and rates), we fitted generalized linear mixed models (GLMM) with a log link, suitable for strictly positive, skewed variables. To account for minor variations in video length, frequency values were standardized as rates per minute. For each variable, duration was calculated as the proportion of its occurrence relative to the total video duration. Each analysis set followed the same model specifications: (1) intercept only; (2) age; (3) behavior code; (4) age + behavior code; and (5) age × condition × behavior code. All models included a random intercept for dyad ID to account for repeated measures within dyads. Model performance was compared using a rank summary of overall fit (Lüdecke et al. 2021), and the highest ranked model was selected. For rates and duration changes in both infant and adult behaviors, all selected models included the age × condition × behavior code interaction with random intercepts. For each selected model, we report marginal and conditional *R* ^2^, unstandardized log‐scale coefficients with standard errors and 95% confidence intervals, and random‐effects estimates. Two‐sided tests were used with *α* = 0.05.

**TABLE 3:** Overview of analytic strategy across study objectives.

Objective	Outcome (dependent variable)	Predictors	Model	Hypothesis
Objective 1: Developmental change in infant and adult behaviors	Behavior rate (per minute) and duration	Age (centered), behavior type, condition, and their interactions	Generalized linear mixed models (GLMM) with random intercepts for dyad	H1a, H1b
Objective 2: Developmental change in multimodal combinations	Frequency of multimodal combinations	Age (centered), behavior type, condition, and their interactions	Generalized linear mixed models (GLMM) with random intercepts for dyad	H2
Objective 3: Associations with communicative and linguistic development	Language outcomes (production and comprehension slopes)	Behavioral growth parameters (rate and duration slopes), language intercepts, and their interaction	Linear regression models	H3

The second plan assessed whether individual behaviors, and their possible multimodal combinations, were associated with infants' communicative and linguistic development (objective 3). For each dyad, we fitted linear models with the behavioral measure as the dependent variable and centered age as the predictor, estimating individual growth parameters: intercepts (estimated value at the mean age) and slopes (rate of change per month). Age was centered at 12.3 months, corresponding to the mean age across the three CDI assessments (9, 12, and 16 months). This procedure was applied to both rate and duration measures for each participant‐behavior combination, and was also used to estimate intercepts and slopes for each language variable (word production and comprehension). All growth parameters were consolidated into a single dataset for subsequent modeling.

To test whether the growth of interactive behaviors was associated with growth in communicative outcomes, we examined the relationship between the slopes of the two language variables (production and comprehension) and the slopes of rate and duration growth for each behavior. For each dependent‐independent variable pair, we fitted a linear regression model including the behavioral slope of interest, the intercept of the corresponding language variable, and their interaction.

Detailed model specifications and full results, including nonsignificant predictors, are reported in the Supplementary Material (Supporting Information S1: Tables S1–S16 and Figure S1), with full production and comprehension models in Supporting Information S1: Tables S15 and S16, respectively, and significant comprehension interactions illustrated in Supporting Information S1: Figure S1. Significant predictors are described in the main text.

All data wrangling and analyses were conducted in R 4.5.0 (R Core Team 2025) using packages tidyverse, broom.mixed, glmmTMB, and performance (Bolker and Robinson 2024; Brooks et al. 2017; Lüdecke et al. 2021; McGillycuddy et al. 2025; Wickham et al. 2019).

## 4 Results

### 4.1 Longitudinal Changes in Infant Behavior

#### 4.1.1 Duration

The model predicting infant behavior duration explained a moderate proportion of variance, with consistent trajectories of change across participants (Table 4). The main effect of age was not significant.

**TABLE 4:** Random effects for models of longitudinal changes in behavior.

Predicted measure	Marginal/Conditional *R* ^2^	*σ* ^2^	*τ*00	*τ*11	*ρ*01	ICC
Duration (infant)	0.51/0.57	0.63	0.03	0.01	−0.41	0.13
Rates (infant)	0.45/0.55	0.47	0.05	0.00	−0.15	0.17
Duration (adult)	0.37/NA	0.60	0.02	0.00	1.00	NA
Rates (adult)	0.30/NA	0.50	0.07	0.00	−1.00	NA
Duration (adult, collapsed)	0.10/0.11	0.69	0.01	NA	NA	0.01
Rates (adult, collapsed)	0.06/0.17	0.59	0.08	NA	NA	0.12
Rates (multimodality)	0.44/0.53	0.43	0.04	0.00	0.17	0.15

Regarding behavioral contrasts, only rhythmic movements with objects showed a significant effect (*B* = 1.79, SE = 0.90, *p* = 0.046). In addition, the interaction between age and spontaneous movements was significant (*B* = −0.80, SE = 0.26, *p* = 0.002), indicating that the duration of spontaneous movements decreased over time relative to the reference behavior (gestures without objects). All other age × behavior code interactions were nonsignificant.

#### 4.1.2 Behavioral Rates

The model predicting infant behavior rates explained a moderate proportion of variance, with limited variability in individual trajectories (Table 4). The main effect of age was not significant (*B* = 0.08, SE = 0.20, *p* = 0.71).

A significant main effect emerged for vocalizations (*B* = 1.78, SE = 0.75, *p* = 0.018), indicating higher rates of vocalization than the reference category (gestures without objects). All other main effects and all age × behavior code interactions were nonsignificant.

### 4.2 Developmental Trajectories of Infant Movement Types

To address Hypothesis 1a, a longitudinal analysis was conducted examining age‐related changes in infants' interactive behaviors, considering movement categories independently of object use and their co‐occurrence with vocalizations. This approach allowed us to isolate whether the type of movement per se (i.e., spontaneous movements, rhythmic movements, and gestures) varied as a function of infant age.

Results showed a significant decrease in spontaneous movements with increasing age (*B* = −0.37, SE = 0.09, *p* < 0.01), alongside a significant increase in rhythmic movements (*B* = 0.25, SE = 0.10, *p* = 0.011). Although gestures emerged descriptively around 8–9 months, their rates remained relatively stable thereafter through 12 months, and no significant linear age‐related change was detected (*B* = 0.09, SE = 0.22, *p* = 0.674).

Figures are intended to provide a descriptive visualization of developmental patterns (e.g., proportions), whereas statistical inferences are based on the model estimates reported in the text and tables. Figure 1 illustrates the proportional distribution of infant movement types across age, showing a predominance of spontaneous movements in the early months and a relative increase in rhythmic movements over time, with gestures appearing more frequently toward the end of the observed period.

**FIGURE 1:**
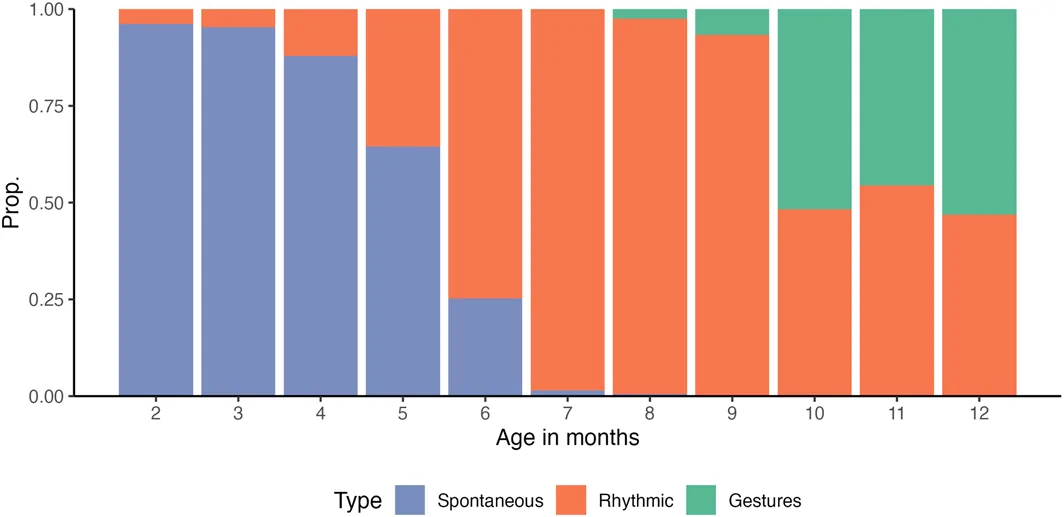
Proportional distribution of infant movement types across age.

These findings partially support Hypothesis 1a, indicating a developmental shift from less organized to more structured forms of movement. Although descriptive patterns suggested an increase in gestures toward the end of the first year (see Figure 1), this change did not reach statistical significance.

### 4.3 Longitudinal Changes in Adult Behavior

#### 4.3.1 Duration

The model predicting adult behavior duration explained a moderate proportion of variance, with consistent trajectories of change across participants (Table 4). A significant negative main effect of age (*B* = −0.17, SE = 0.03, *p* < 0.001) indicated that the duration of the reference behavior (Level 1 of distal object‐use demonstrations) decreased over time.

Regarding behavioral contrasts, several significant differences emerged relative to the reference category. Lower durations were observed for Level 3 distal object‐use demonstrations (*B* = −0.42, SE = 0.15, *p* = 0.005), Level 4 demonstrations (*B* = −1.09, SE = 0.19, *p* < 0.001), non‐rhythmic touch with objects (*B* = −1.54, SE = 0.15, *p* < 0.001), rhythmic touch without object (*B* = −0.50, SE = 0.15, *p* = 0.001) and rhythmic touch with objects (*B* = −0.31, SE = 0.14, *p* = 0.022).

Significant interactions with age indicated that the duration of Level 2 (*B* = 0.12, SE = 0.04, *p* = 0.004), Level 3 (*B* = 0.19, SE = 0.04, *p* < 0.001), and non‐rhythmic touch without objects (*B* = 0.22, SE = 0.04, *p* < 0.001) increased over time relative to the reference behavior. All other age × behavior code interactions were nonsignificant.

#### 4.3.2 Behavioral Rates

Figure 2 shows the monthly proportions of adult distal object‐use demonstration levels. The relative rates of Level 1 steadily decrease with age, while Levels 2, 3, and 4 show progressive increases across the first year. Level 1 is the most frequent category overall, and Levels 2, 3 and 4, though less common, gain ground with age. This descriptive pattern aligns with the model estimates, which revealed a negative main effect of age for the reference behavior (Level 1), and positive age‐by‐level interactions for Level 2 and Level 3.

**FIGURE 2:**
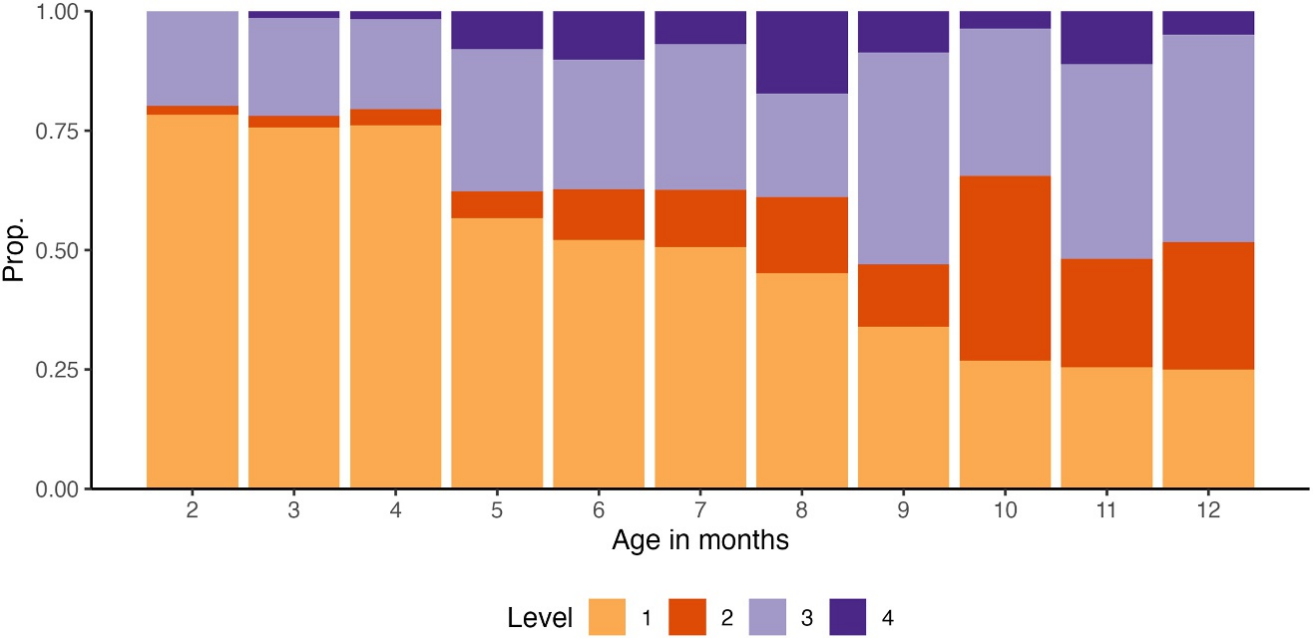
Proportional distribution of adult distal object‐use demonstration levels across infant age. The levels used to differentiate the complexity of adults’ object‐use demonstrations were: Level 1: non‐rhythmic or unconventional handling; Level 2: striking the object against a surface; Level 3: conventional rhythmic use (e.g., shaking the maraca); Level 4: symbolic use (e.g., using it as a microphone).

The model predicting adult behavior rates explained a modest proportion of variance, with minimal variability in individual trajectories (Table 4).

A significant negative main effect of age (*B* = −0.14, SE = 0.02, *p* < 0.001) indicated that the rates of the reference behavior decreased over time.

Regarding main effects of behavior, several categories showed significantly lower rates compared to the reference. Specifically, Level 2 (*B* = −0.80, SE = 0.15, *p* < 0.001), Level 3 (*B* = −0.32, SE = 0.12, *p* = 0.009), Level 4 (*B* = −1.05, SE = 0.18, *p* < 0.001) distal demonstrations occurred less frequently than the reference (Level 1). Similarly, non‐rhythmic touch without objects (*B* = −0.30, SE = 0.12, *p* = 0.017), non‐rhythmic touch with objects (*B* = −1.01, SE = 0.13, *p* < 0.001), and rhythmic touch without objects (*B* = −0.52, SE = 0.13, *p* < 0.001) also showed lower frequencies than the reference.

Significant interactions with age showed that the rates of Level 2 (*B* = 0.29, SE = 0.04, *p* < 0.001), Level 3 (*B* = 0.16, SE = 0.04, *p* < 0.001), and non‐rhythmic touch without objects (*B* = 0.17, SE = 0.04, *p* < 0.001) increased over time relative to the reference (Level 1). All other interactions were nonsignificant.

### 4.4 Developmental Trajectories of Adult Interaction Strategies Across Infant Age

To directly address Hypothesis 1b, a longitudinal analysis was conducted examining changes over time as a function of infants' age. For this purpose, interaction behaviors were grouped according to their proximity to the infant's body into two broad categories: distal strategies, including Levels 1 to 4 of distal demonstrations, and proximal strategies, comprising all rhythmic and non‐rhythmic touches, both with and without objects. This classification enabled us to capture shifts in the spatial organization of adult–infant interaction across development.

Figure 3 illustrates the longitudinal trajectories of proximal and distal strategies across infant age. This figure reflects model‐based trajectories expressed as rate per minute and should be interpreted in conjunction with the statistical estimates reported above. For clarity, the figure aggregates across subcategories that are modeled separately in the statistical analyses, and reflects a separate model using aggregated proximal and distal categories. Results on proportional duration showed a similar pattern.

**FIGURE 3:**
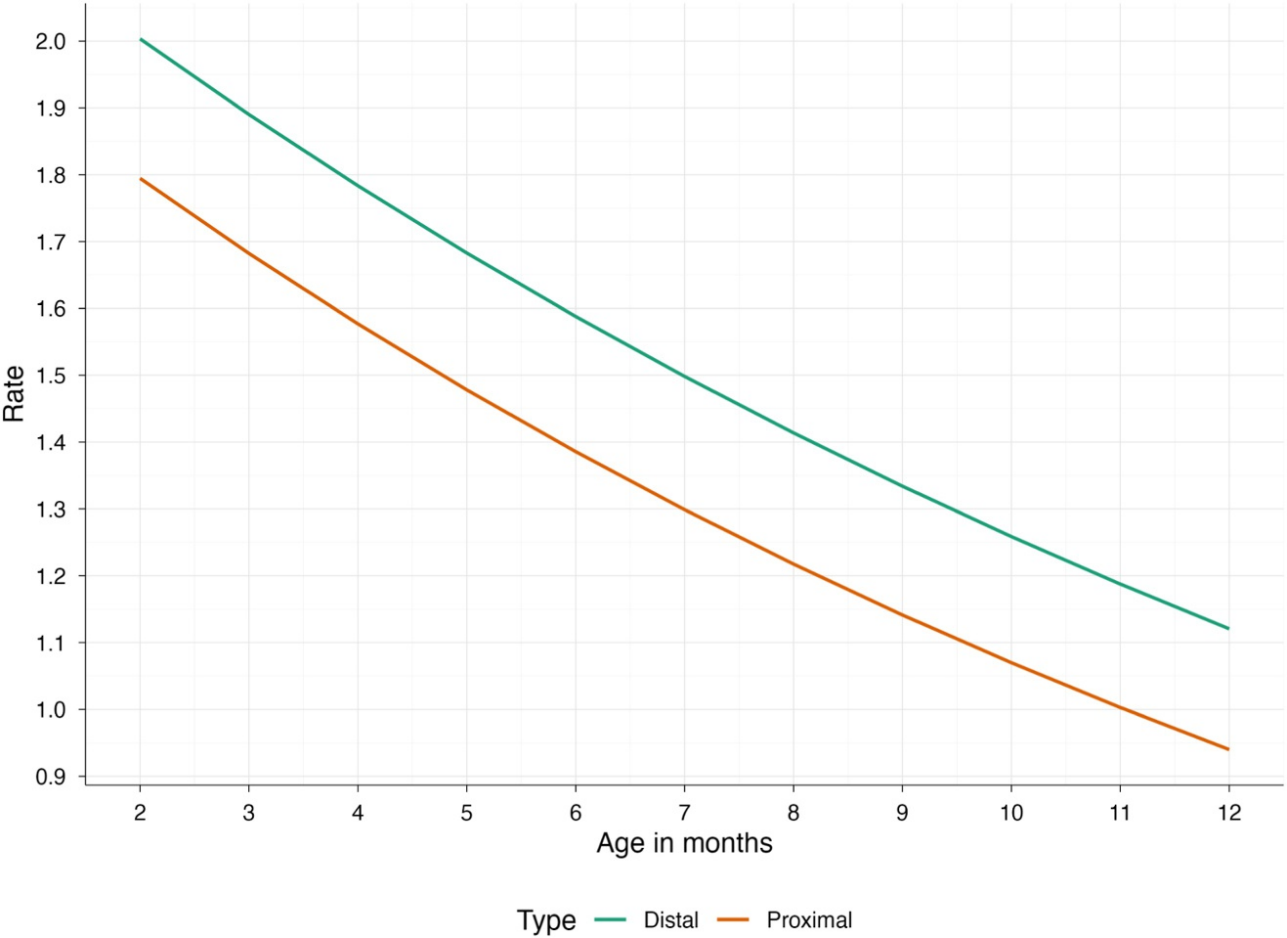
Adult proximal and distal interaction strategies across infant age.

For duration of behaviors (expressed as proportional time), results showed a significant main effect of age, indicating that the proportional duration of interaction strategies decreased with increasing infant age (*B* = −0.08, SE = 0.01, *p* < 0.001), averaged across proximal and distal strategies. Proximal strategies were associated with significantly lower proportional duration compared to distal strategies (*B* = −0.21, SE = 0.08, *p* = 0.012).

For behavior rate, age was again a significant predictor, with behavior rates decreasing as infant age increased (*B* = −0.06, SE = 0.01, *p* < 0.001). Proximal strategies showed a lower rate compared to distal strategies (*B* = −0.15, SE = 0.08, *p* = 0.049).

Overall, both measures showed a similar pattern, with a general decrease in adult interaction strategies as infant age increased. No interaction between age and strategy type was observed, indicating that this decrease applied similarly to both proximal and distal strategies. These findings partially support Hypothesis 1b, as adult interaction strategies showed age‐related changes; however, contrary to expectations, both proximal and distal strategies decreased with infant age rather than showing a shift from proximal to distal strategies.

### 4.5 Multimodality in Infant Behavior

To address Hypothesis 2, a longitudinal model was conducted to examine age‐related changes in the rates of multimodal combinations in infants' behaviors. The model explained a moderate proportion of variance, with consistent trajectories of change across participants (Table 4).

A significant negative main effect of age (*B* = −0.37, SE = 0.09, *p* < 0.001) indicated that the rate of the reference behavior (spontaneous movements without objects) decreased over time. Among the main effects, multimodal combinations involving vocalizations were associated with lower frequencies (*B* = −1.01, SE = 0.43, *p* = 0.018).

Significant two‐way interactions showed that the rates of rhythmic movements (*B* = 0.25, SE = 0.10, *p* = 0.011), and behaviors involving object use (*B* = 0.29, SE = 0.15, *p* = 0.049) increased over time relative to the reference levels. Additionally, rhythmic movements combined with object use occurred at higher rates over time (*B* = 1.04, SE = 0.50, *p* = 0.039) compared to the reference combination (spontaneous movements without object use). All other main effects and higher‐order interactions were nonsignificant.

Figures 4 and 5 illustrate the proportion of multimodal combinations involving object use and vocalizations, respectively, across infant age. Combinations involving object use increased over time, becoming predominant from around 5 months onward, whereas combinations involving vocalizations remained relatively stable, with no marked age‐related changes. This pattern is consistent with the model estimates, which indicated a significant positive effect of age for combinations with object use (*B* = 0.29, SE = 0.15, *p* = 0.05) but no significant change for those including vocalization (*B* = 0.02, SE = 0.13, *p* = 0.86).

**FIGURE 4:**
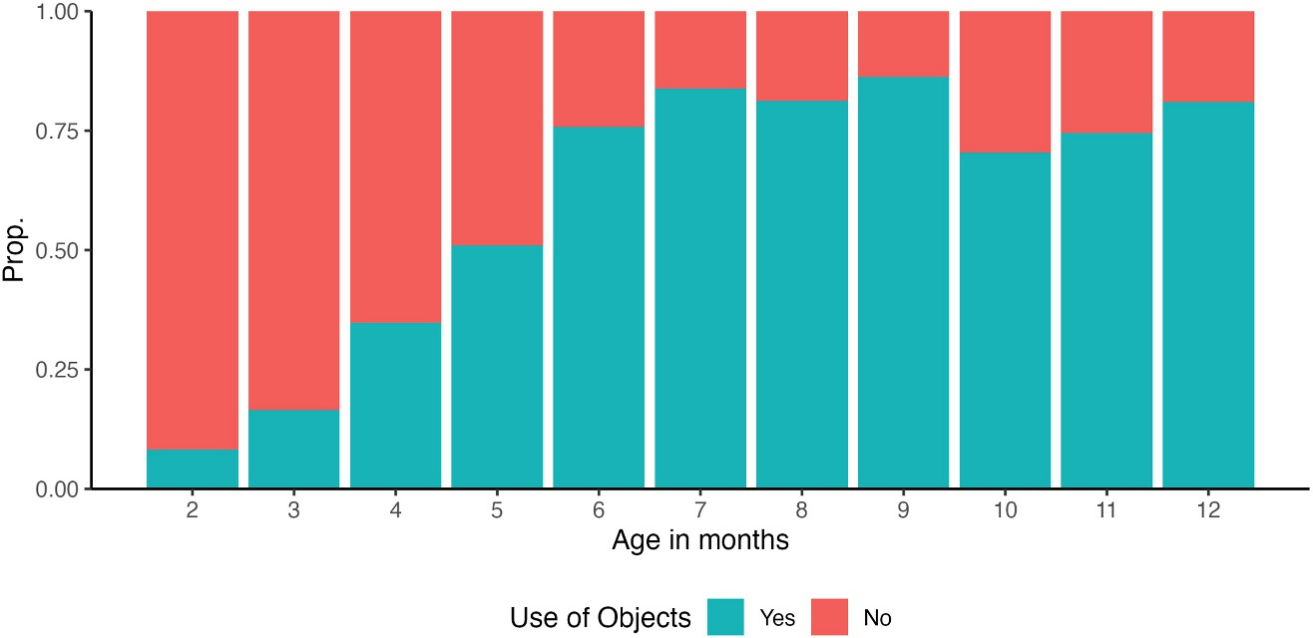
Proportion of multimodal combinations involving object use across infant age.

**FIGURE 5:**
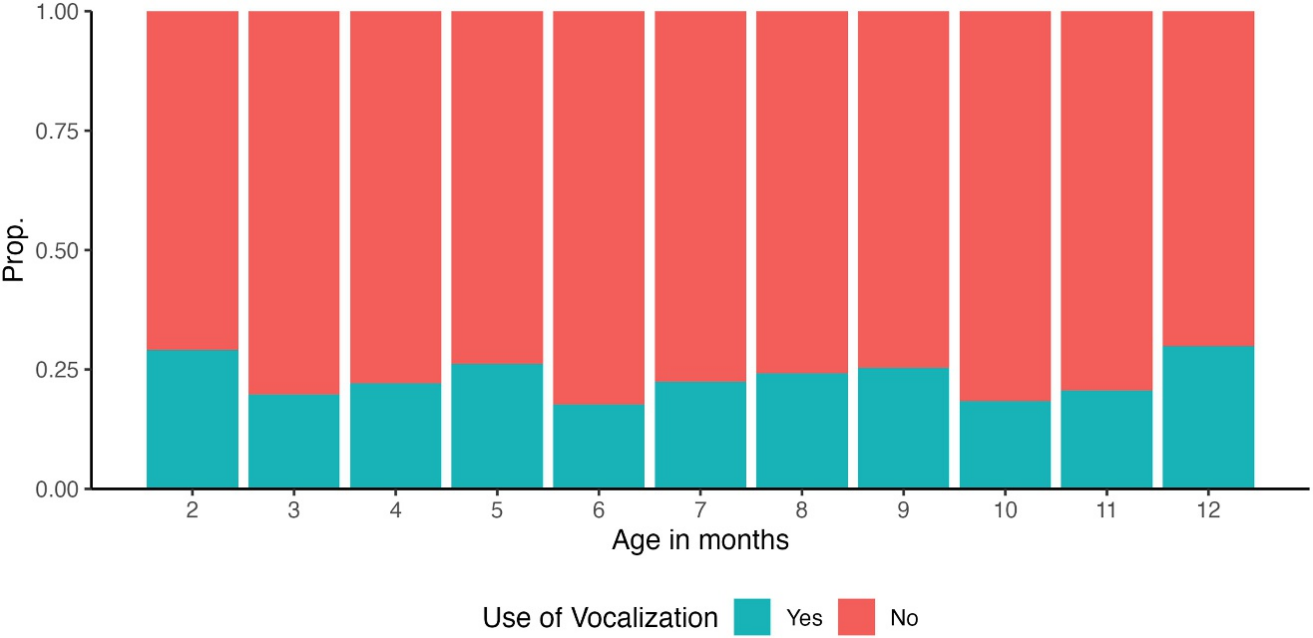
Proportion of multimodal combinations involving vocalization across infant age.

These findings partially support Hypothesis 2, as infant behaviors involving object use increased with age, whereas those involving vocalizations remained stable during the first year.

### 4.6 Associations With Measures of Infants' Communicative Development

To address Hypothesis 3, a series of longitudinal models were conducted to examine whether individual differences in adults' and infants' interactive behaviors were associated with growth trajectories in communicative and linguistic development from 9 to 16 months, as indexed by the slopes of word production and comprehension.

Overall, models predicting either productive vocabulary or comprehension were significant, although several behavioral measures did not reach significance (See Supporting Information S1: S15 and S16). In most significant models, there was a significant positive main effect of the level of the corresponding language measure at the mean assessment age (B range = 0.215–0.529 for production models; 0.171–0.387 for comprehension models).

For word production, significant interaction effects emerged between behavioral measures and the estimated level of word production at the mean age of CDI assessment (12.3 months) (Table 5). These included the proportional duration of infants' rhythmic movements with objects, the rates of infant behaviors aggregated based on object use and absence of vocalizations, and the rates of adults' Level 4 distal demonstrations. All interaction effects were negative (see Table 5), indicating that the association between behavioral change and production growth became increasingly negative as estimated production level at the mean assessment age (12.3 months) increased. At lower production levels, this association was weak or close to zero, whereas at higher levels it was more strongly negative (Figure 6).

**TABLE 5:** Significant interaction effects of behavior variables with level of word production at mean age.

Behavior variables	*B*	SE	CI	*p*
Proportional duration of infants' rhythmic movements with objects	−22.554	8.343	[−44.001, −1.107]	0.043
Rates of adults' level 4 of object use demonstration (symbolic uses)	−4.504	1.021	[−7.128, −1.88]	0.007
Infant categories aggregated based on the presence of object use	−4.200	1.007	[−6.789, −1.612]	0.009
Infant categories aggregated based on the absence of vocalizations	−7.733	1.890	[−12.593, −2.874]	0.009

**FIGURE 6:**
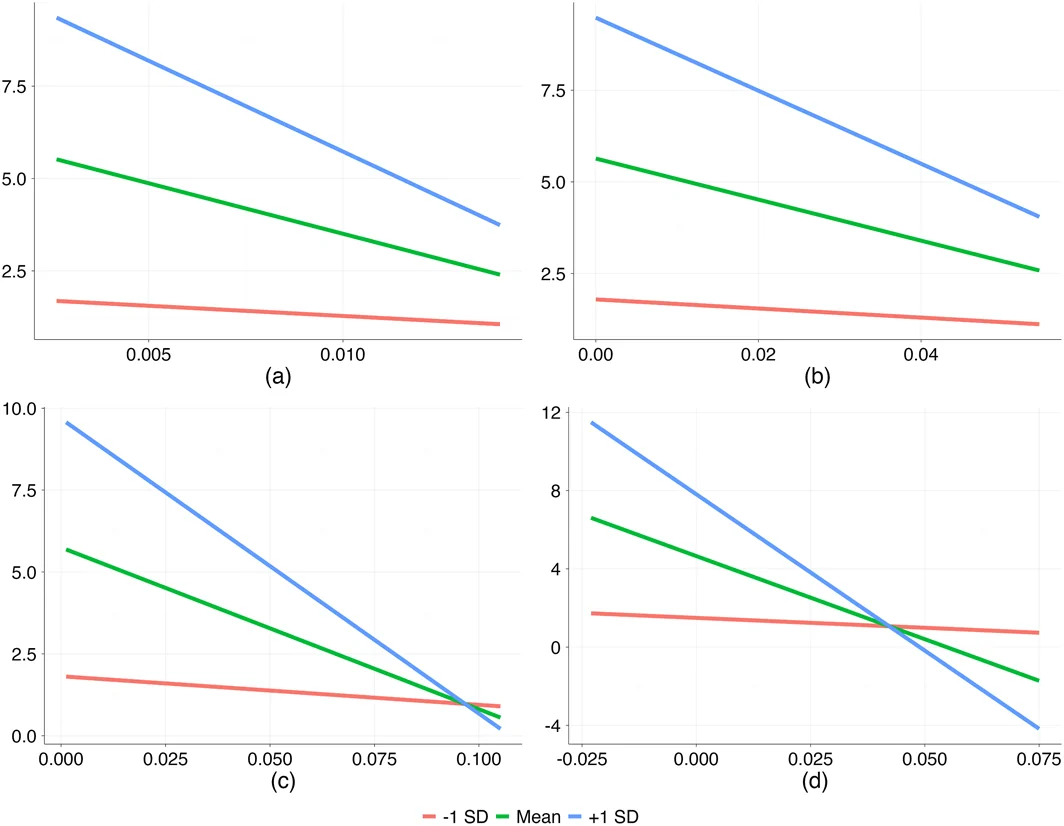
Significant interaction effects of behavior variables with estimated word production level at 12.3 months. Lines represent −1 SD (red), mean (green), and +1 SD (blue) of the moderator, defined as the intercept of estimated word production level at 12.3 months (mean CDI assessment age). Y‐axis: word production slope. X‐axis: behavioral slope. (a) Adults' Level 4 object‐use demonstrations (symbolic uses). (b) Proportional duration of infants' rhythmic movements with objects. (c) Infant categories based on the presence of object use. (d) Infant categories based on the absence of vocalizations.

To facilitate the interpretation of the significant interaction effects reported in Table 5, Figure 6 illustrates the predicted relationships between behavioral slopes and word production slope at different levels of the moderator, defined as the intercept of word production at the mean age of the language assessments.

For comprehension, significant effects of behavioral trajectories were observed for infants' spontaneous movements without vocalizations, infant behaviors aggregated based on the absence of object use, and the proportional duration of adults' Level 4 object‐use demonstrations (symbolic uses) (Table 6). For the two infant behavioral measures, these effects were further qualified by significant interactions with estimated comprehension level at 12.3 months, described below.

**TABLE 6:** Significant main effects of behavior measures on comprehension.

Behavior variables	*B*	SE	CI	*p*
Duration of adults' level 4 of object use demonstration (symbolic uses)	2564.016	933.104	[165.397, 4962.635]	0.040
Rates of infants' spontaneous movements in the absence of vocalizations	43.185	13.760	[7.813, 78.557]	0.026
Infant categories aggregated based on the absence of object use	235.118	72.477	[48.809, 421.427]	0.023

In addition, significant negative interaction effects were found between behavioral trajectories and estimated comprehension level at 12.3 months for infants' spontaneous movements in the absence of object use, spontaneous movements in the absence of vocalizations, and infant behaviors aggregated based on the absence of object use (Table 7). These interactions indicated that the association between behavioral change and comprehension slope varied as a function of comprehension level: it was positive at lower estimated comprehension levels, close to zero around the mean, and negative at higher levels.

**TABLE 7:** Significant interaction effects of behavior measures with level of comprehension at mean age.

Behavior variables	*B*	SE	CI	*p*
Rates of infants' spontaneous movements in the absence of object use	−0.658	0.233	[−1.256, −0.061]	0.037
Rates of infants' spontaneous movements in the absence of vocalizations	−0.595	0.180	[−1.058, −0.132]	0.021
Infant categories aggregated on the absence of object use	−3.365	1.135	[−6.284, −0.447]	0.031

Overall, these findings partially support Hypothesis 3 by showing behavioral trajectories were associated with individual language‐growth trajectories, with distinct moderation patterns for comprehension and production as a function of estimated language level at 12.3 months.

## 5 Discussion

Our results indicate that, over the first year of life, infants and caregivers progressively adjust their actions and signals to construct shared meanings. Communication during this period becomes an increasingly complex system in which expressive modalities—such as gaze, gesture, object manipulation, vocalization, and touch—are integrated into progressively more structured patterns (Fogel et al. 2006; Iverson 2010). From a multimodal and triadic perspective, these changes emerge from the dynamic interplay between child, adult, and material context rather than within a single channel (Mendoza‐García and Moreno‐Núñez 2023). Within this framework, our findings show that infant and adult behaviors follow distinct developmental trajectories, and that associations between behavioral and language‐growth trajectories vary according to infants' estimated language level at the mean assessment age.

For infants, the most marked age‐related change occurred in the motor domain: the duration of spontaneous movements steadily declined over the first year. In relation to Hypothesis 1a, results showed that age‐related changes depended on the level of behavioral aggregation. While models examining more fine‐grained behavioral categories—specifically distinguishing infant behaviors based on object use—did not capture significant age‐related effects, analyses grouping behaviors by movement type independently of object use revealed clear and statistically significant developmental patterns, with spontaneous movements decreasing and rhythmic movements increasing with age. At the same time, no significant age‐related changes were observed for gestures, although they became more prominent descriptively toward the end of the first year.

Based on the descriptive patterns observed in Figure 1, rhythmic movements appear to increase during the first months, reaching a peak around 6 months before declining, whereas gestures first emerge around 8–9 months and remain relatively stable thereafter, a pattern consistent with Azagra et al. (2026). While within‐subject variance is high in this sample (Table 4), between‐subject differences align with longitudinal evidence describing a reorganization of the expressive repertoire across the first year: an early decline in spontaneous movements, a mid‐year peak in rhythmic actions, and a later rise in gestures, while stable multimodal patterns remain rare (Azagra et al. 2026). Taken together, these findings partially support Hypothesis 1a, suggesting a developmental shift from less organized to more structured forms of movement. This shift may reflect a reallocation of resources from non‐communicative spontaneous activity toward more intentionally social behaviors—such as shared object manipulation and rhythmic routines—which may reflect a sensorimotor pathway that supports the emergence of coordinated communication.

In relation to Hypothesis 1b, the expected shift from proximal to distal interaction strategies was not supported. Instead, adult behaviors followed a similar trajectory over time, with both proximal and distal strategies decreasing. The absence of an interaction between age and strategy type indicates that this reduction occurred uniformly across both forms of interaction. This pattern suggests a general decline in adult‐initiated interventions as infants develop greater communicative and interactive capacities. As infants become more active participants, caregivers may progressively allow more space for the child's initiatives, reducing the need for continuous scaffolding through touch or object‐based demonstrations.

This interpretation is consistent with previous work showing that adult‐initiated physical contact tends to decrease as infants grow and become more independent (Murillo et al. 2025), and more broadly with research highlighting that caregiver behaviors adapt dynamically to infants' developmental changes (Dimitrova and Moro 2013; Mendive et al. 2013). From this perspective, the present results suggest that such adaptive processes extend across different types of interaction strategies, rather than reflecting a simple substitution of proximal by distal forms.

Importantly, beyond this overall reduction, the findings also point to a qualitative reorganization within distal object‐use demonstrations. Simpler forms (Level 1) became less frequent, and more complex forms (Levels 2, 3, and 4) gained relative prominence over time. This pattern points to a shift toward more elaborated uses of objects when these strategies are deployed, even as their overall frequency declines.

An alternative, non‐mutually exclusive explanation is that the observed decrease reflects a shift toward other communicative modalities not captured in the present analysis. As infants grow older, caregivers may increasingly rely on gestures, verbal input, or richer multimodal configurations, which could replace or complement the proximal and distal strategies examined here.

In this context, touch—although decreasing in frequency in our data—may still play an important regulatory and supportive role, for example in guiding attention, supporting exploration, or marking co‐presence (Hertenstein 2002; Serra et al. 2020), even if its relative use diminishes as other communicative channels become more prominent within an increasingly multimodal system.

These developmental changes in infant and adult repertoires were also reflected in multimodal co‐occurrences. The results were partially consistent with Hypothesis 2, showing a differentiated pattern depending on the type of multimodal combination. Combinations integrating rhythmic, object‐related movements increased significantly with age, whereas those involving vocalization remained stable, with no significant age‐related changes. This supports the idea that perceptually salient sensorimotor coordinations (e.g., rhythmically shaking an object while looking at the adult) consolidate earlier than the stable integration of the vocal channel (Azagra et al. 2026; Murillo et al. 2018).

Longitudinal evidence shows that such rhythmic multimodal movements at 9–10 months predict the emergence of proximal deictic gestures (Murillo et al. 2021) and elicit referential responses from adults (Moreno‐Núñez et al. 2021). The rarity of gesture‐vocalization combinations before 12 months, despite their early emergence, is consistent with the view that their intentional coordination becomes more firmly established toward the end of the first year and expands in the second (Esteve‐Gibert and Prieto 2014). In our data, vocalizations occurred more frequently as stand‐alone acts than as coordinated combinations with objects, suggesting that early vocal expression is often independent from other modalities. Even so, early gesture‐vocalization combinations may hold predictive value for later language development (Igualada et al. 2015).

Viewed together, the longitudinal increase in rhythmic movements and object engagement may reflect an emerging shift toward intentional triadic reference, underpinned by advances in joint attention and shared object‐focused action. This is consistent with classical evidence situating the transition from dyadic to triadic interactions between 9 and 12 months (Carpenter et al. 1998; Colonnesi et al. 2010). Contrary to our expectations, we did not observe a significant longitudinal increase in infants' gestures in this age range, possibly because gestures emerged relatively late in the period examined and remained relatively stable thereafter. Further changes in gesture production may become more evident during the second year of life, which was not examined in the present study (Liszkowski et al. 2012; Salo et al. 2018). Instead, the observed growth in rhythmic, object‐related multimodal coordination suggests that early triadic episodes are initially scaffolded by temporally and materially structured actions before the stable production of intentional gestures. By contrast, vocalizations and gestures without objects showed comparatively stable trajectories across the observed period, indicating that the developmental changes observed for rhythmic and object‐related behaviors did not extend uniformly across modalities. Importantly, even before infants produce ostensive gestures themselves, adults contribute to constructing triadic episodes by following and expanding the infant's focus of attention (Alessandroni et al. 2020; Mendoza‐García and Moreno‐Núñez 2023; Striano and Bertin 2005). Thus, the significant increase in rhythmic movements and in multimodal combinations involving object use can be seen as an early, visible manifestation of an interactive process co‐constructed from the earliest months through adult responsiveness (Duque‐Fernández and Moreno‐Núñez 2022).

Multimodality not only characterizes infant behavior but also shapes interaction: infants' multimodal signals tend to elicit richer, more contingent adult responses (Van der Klis et al. 2023). For instance, when an infant rhythmically shakes a rattle while looking at the adult, the adult often responds by integrating multiple modalities (speech, object manipulation, touch). Such coordinated behaviors have been shown to occur within joint attention episodes and to support longer periods of sustained attention to objects (Suárez‐Rivera et al. 2019; Yu and Smith 2016), highlighting the role of multimodal parent–infant coordination in structuring infants' engagement with the environment. These interactional contexts, in turn, may create favorable conditions for early word–object mapping (Gogate et al. 2000). In line with this, our findings suggest that these multimodal, object‐centered interactions not only structure infants' attention but also show systematic developmental changes and are linked to the emergence of early communicative behaviors across the first year.

In relation to Hypothesis 3, from a longitudinal perspective, our analyses showed that associations between nonverbal behavioral trajectories and language growth across 9–16 months varied as a function of infants' estimated comprehension and production levels at the mean CDI assessment age (12.3 months). This pattern is consistent with longitudinal evidence showing continuity between early communicative skills and later linguistic development (Caselli et al. 2012; Colonnesi et al. 2010), while adding that these relations are not uniform across behavioral trajectories or language levels. For word production, the association between change in several nonverbal behaviors and vocabulary growth became increasingly negative at higher estimated production levels, whereas at lower levels these associations were weak or close to zero. These findings underscore the value of behavioral observation alongside parental reports, as different forms of nonverbal participation may capture aspects of communicative development that are not fully reflected in vocabulary measures alone.

Behaviors without object use were also associated with verbal comprehension, with the direction of this association varying according to estimated comprehension level at 12.3 months: it was positive at lower levels, close to zero around the mean, and negative at higher levels. These behaviors may reflect moments when the infant is free from manipulating the object, allowing greater attention to adult demonstrations, verbal explanations, and other cues. This configuration may facilitate receptive language development by freeing attentional resources for linguistic input. Importantly, however, the absence of object use should not necessarily be equated with an entirely unimodal interaction, as other communicative resources may still be available within the ongoing exchange.

Similarly, spontaneous movements without vocalization—largely automatic motor patterns linked to early neural maturation (Prechtl and Hopkins 1986)—showed a comparable interaction with comprehension level, despite decreasing clearly across the first year. Notably, this finding may appear counterintuitive, as these movements are typically considered non‐communicative behaviors, lacking an explicit intent to convey meaning to others. Rather than suggesting a uniformly positive role of spontaneous movements in language development, this association may reflect broader developmental reorganization within the motor and communicative system. This interpretation is consistent with developmental cascade accounts (Gonzalez et al. 2019; Schneider and Iverson 2022; Thelen and Smith 2006; Walle and Campos 2014), according to which changes in one developmental domain may be associated with changes in another. More generally, these findings suggest that developmental relevance does not depend simply on the number of modalities simultaneously involved: behavioral configurations lacking one coded modality may still provide information about changes in the broader communicative system.

A further unexpected finding was the absence of a reliable association between infants' gesture trajectories and language growth, given the well‐established links between early gesture and subsequent lexical development (Colonnesi et al. 2010; Igualada et al. 2015). In the present study, however, gestures emerged relatively late in the observation period, around 8–9 months, and remained comparatively stable thereafter through 12 months. This left a relatively restricted developmental window in which individual differences in gesture trajectories could emerge. Together with the small sample, this may have limited our ability to detect associations with language growth. Thus, this null finding should not be interpreted as evidence against the role of gesture in language development, but rather as potentially reflecting the developmental period captured in the present study.

Adult behaviors were also related to infants' communicative development, with distinct patterns for comprehension and production. Across outcomes, symbolic object‐use demonstrations (Level 4) emerged as the only adult behavior consistently associated with language development. For comprehension, the proportional duration of symbolic demonstrations showed a positive association with the comprehension trajectory. For production, by contrast, symbolic demonstrations interacted with estimated production level at 12.3 months: the association between change in symbolic demonstrations and production growth was weak or close to zero at lower production levels and became more negative at higher levels.

One possible explanation is that symbolic object use creates communicatively rich and structured interactional contexts, in which language, shared attention, and representational play are integrated. These contexts may support infants' understanding of linguistic input and scaffold their participation in interaction. Importantly, this relationship may be bidirectional: caregivers may increasingly use symbolic actions as infants show more advanced communicative capacities, while these same interactions may in turn contribute to the contexts in which communicative development unfolds.

No other adult behaviors showed reliable associations with language trajectories. Tactile behaviors, although following their own developmental trajectory, were not directly associated with language growth in this study. Taken together, these findings partially support Hypothesis 3, indicating that specific infant and adult behavioral trajectories were associated with language growth and that some of these relationships varied according to infants' estimated language level at the mean assessment age.

Taken together, these results point to a set of coordinated developmental shifts across infant and caregiver behaviors that are not fully captured by individual analyses. To integrate these findings, Figure 7 presents a conceptual model summarizing the patterns observed in our data regarding the developmental trajectories of infant and caregiver behaviors across the first year of life. Based on simulated data to support the theoretical proposal, the model shows a shift in infants from predominantly spontaneous movements toward more structured rhythmic and object‐related actions, followed by the gradual emergence of gestures, while vocalizations remain relatively stable. In parallel, caregivers reduce the frequency of their interventions but increasingly rely on more complex and symbolic object‐use demonstrations as infants grow older. These patterns suggest a dynamic process of mutual adaptation between infants and caregivers, offering a coherent framework to interpret the observed developmental changes.

**FIGURE 7:**
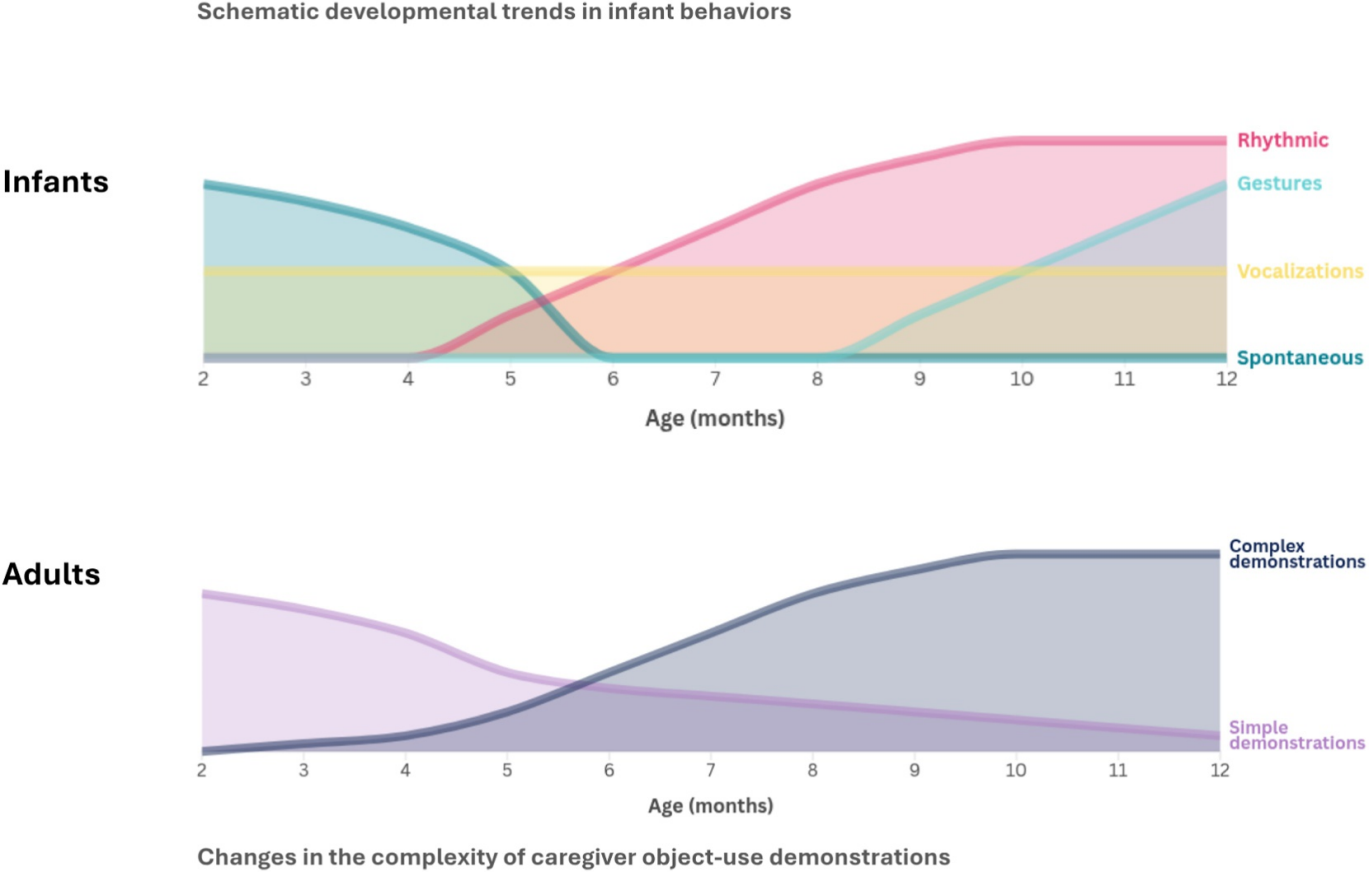
Schematic developmental trends in infant and adult behaviors.

Although the sample was small, the longitudinal design and multimodal analysis enabled the identification of patterns rarely documented in previous research, generating new hypotheses about the development of triadic communication in the first year. In addition, the participating families shared a relatively similar cultural background, which may limit the generalizability of the findings, as caregiver–infant interaction styles can vary across cultural contexts.

Future studies could extend the age range to 24 months to capture the consolidation of more complex multimodal integrations (Capirci et al. 2005; Rohlfing et al. 2017), differentiate gesture functions (e.g., imperative vs. declarative) (Moreno‐Núñez et al. 2020), and analyze adult behaviors within a fully multimodal framework integrating speech, gestures, prosody, and touch (Kosie and Lew‐Williams 2024). Examining adult‐infant temporal and semantic contingency will also be important (Koterba and Iverson 2009; Yu and Smith 2016), as will applying this approach to atypical developmental populations to explore the universality of these trajectories, building on emerging work examining parent–child interaction in children with diverse developmental profiles (e.g., Mendoza‐García et al. 2025; Perkovich et al. 2022).

Overall, our findings support the view that early communicative development is a dynamic, multimodal system in which motor, vocal, and material channels are bidirectionally modulated between infant and adult. Insights into the role of gestures and motor behaviors, the adaptive function of touch, the sequence of multimodal emergence, and the conditional associations between nonverbal behavior and language growth contribute to a deeper understanding of the first stages of triadic communication. Taken together, the findings suggest that infants' engagement with objects is already integrated into communicative exchanges during the first months of life, pointing to the role of material involvement in the early emergence of triadic communication and shared intersubjectivity.

## Author Contributions

**Ana Mendoza‐García:** conceptualization, investigation, data curation, validation, writing – original draft, writing – review and editing, methodology. **Ana Moreno–Núñez:** conceptualization, investigation, validation, supervision, funding acquisition, project administration, writing – review and editing, methodology. **Andrés Aparicio:** data curation, formal analysis, visualization, writing – review and editing.

## Ethics Statement

All procedures were approved by the Research Ethics Committee of the Universidad Autónoma de Madrid (CEI‐103–1967), and informed consent was obtained from all families in accordance with the ethical standards outlined in the Declaration of Helsinki.

## Conflicts of Interest

The authors declare no conflicts of interest.

## Supporting information


Supporting Information S1

## Data Availability

The datasets used in this study, as well as the data analysis scripts, will be made publicly available via the Open Science Framework (OSF) upon publication of the article. Due to ethical constraints related to the use of video material involving infants, raw video recordings cannot be shared. All shared materials will be fully anonymized to ensure participant confidentiality.
