# Supplementary material for: Multimodal Dynamics in Early Triadic Interactions at Home: A Longitudinal Analysis Throughout Infants' First Year of Life
Source: Infancy. 2026 Nov 13;31(6):e70136. doi: 10.1111/infa.70136 (PMC13762369; doi:10.1111/infa.70136)
Supplement: Supplementary file 1 [file INFA-31-0-s001.docx]

**Supplementary Material**

***Multimodal dynamics in early triadic interactions at home: A longitudinal analysis throughout infants’ first year of life***

**Variable codes and nomenclature**

Infant behaviors

child_behaviour_duration_proportional_GES: Infant gestures without object use – proportional duration.

child_behaviour_duration_proportional_GES_OBJ: Infant gestures with object use – proportional duration.

child_behaviour_duration_proportional_M_ESP: Infant spontaneous movements without object use – proportional duration.

child_behaviour_duration_proportional_M_ESP_OBJ: Infant spontaneous movements with object use – proportional duration.

child_behaviour_duration_proportional_M_RIT: Infant rhythmic movements without object use – proportional duration.

child_behaviour_duration_proportional_M_RIT_OBJ: Infant rhythmic movements with object use – proportional duration.

child_behaviour_duration_proportional_VOC: Infant vocalizations – proportional duration.

child_behaviour_rate_GES: Infant gestures without object use – rate.

child_behaviour_rate_GES_OBJ: Infant gestures with object use – rate.

child_behaviour_rate_M_ESP: Infant spontaneous movements without object use – rate.

child_behaviour_rate_M_ESP_OBJ: Infant spontaneous movements with object use – rate.

child_behaviour_rate_M_RIT: Infant rhythmic movements without object use – rate.

child_behaviour_rate_M_RIT_OBJ: Infant rhythmic movements with object use – rate.

child_behaviour_rate_VOC: Infant vocalizations – rate.

behaviour_rate_ESP_NOVOC: Infant spontaneous movements without object use or vocalization – rate.

behaviour_rate_ESP_OBJ_NOVOC: Infant spontaneous movements with object use, without vocalization – rate.

behaviour_rate_ESP_OBJ_VOC: Infant spontaneous movements with object use and vocalization – rate.

behaviour_rate_ESP_VOC: Infant spontaneous movements without object use, with vocalization – rate.

behaviour_rate_GES_NOVOC: Infant gestures without object use or vocalization – rate.

behaviour_rate_GES_OBJ_NOVOC: Infant gestures with object use, without vocalization – rate.

behaviour_rate_GES_OBJ_VOC: Infant gestures with object use and vocalization – rate.

behaviour_rate_GES_VOC: Infant gestures without object use, with vocalization – rate.

behaviour_rate_RIT_NOVOC: Infant rhythmic movements without object use or vocalization – rate.

behaviour_rate_RIT_OBJ_NOVOC: Infant rhythmic movements with object use, without vocalization – rate.

behaviour_rate_RIT_OBJ_VOC: Infant rhythmic movements with object use and vocalization – rate.

behaviour_rate_RIT_VOC: Infant rhythmic movements without object use, with vocalization – rate.

mov_behaviour_rate_ESPON: Infant spontaneous movements – rate (aggregated across object use and vocalization).

mov_behaviour_rate_RITM: Infant rhythmic movements – rate (aggregated across object use and vocalization).

mov_behaviour_rate_GESTOS: Infant gestures – rate (aggregated across object use and vocalization).

obj_behaviour_rate_SI: Infant behaviors with object use – rate.

obj_behaviour_rate_NO: Infant behaviors without object use – rate.

voc_behaviour_rate_SI: Infant behaviors with vocalization – rate.

voc_behaviour_rate_NO: Infant behaviors without vocalization – rate.

Adult behaviors

adult_behaviour_duration_proportional_DEM_1: Adult Level 1 object-use demonstrations - proportional duration.

adult_behaviour_duration_proportional_DEM_2: Adult Level 2 object-use demonstrations - proportional duration.

adult_behaviour_duration_proportional_DEM_3: Adult Level 3 object-use demonstrations - proportional duration.

adult_behaviour_duration_proportional_DEM_4: Adult Level 4 symbolic object-use demonstrations - proportional duration.

adult_behaviour_duration_proportional_NRT_DIR: Adult non-rhythmic touch without object use - proportional duration.

adult_behaviour_duration_proportional_NRT_OBJ: Adult non-rhythmic touch with object use - proportional duration.

adult_behaviour_duration_proportional_RT_DIR: Adult rhythmic touch without object use - proportional duration.

adult_behaviour_duration_proportional_RT_OBJ: Adult rhythmic touch with object use - proportional duration.

adult_behaviour_rate_DEM_1: Adult Level 1 object-use demonstrations - rate.

adult_behaviour_rate_DEM_2: Adult Level 2 object-use demonstrations - rate.

adult_behaviour_rate_DEM_3: Adult Level 3 object-use demonstrations - rate.

adult_behaviour_rate_DEM_4: Adult Level 4 symbolic object-use demonstrations - rate.

adult_behaviour_rate_NRT_DIR: Adult non-rhythmic touch without object use - rate.

adult_behaviour_rate_NRT_OBJ: Adult non-rhythmic touch with object use - rate.

adult_behaviour_rate_RT_DIR: Adult rhythmic touch without object use - rate.

adult_behaviour_rate_RT_OBJ: Adult rhythmic touch with object use - rate.

**Section S1. Longitudinal models of infant and adult behavior**

**Table S1. Duration of infant behavior (Fixed effects)**

| Predictors | Estimates | std. Error | CI | Statistic | p |
| --- | --- | --- | --- | --- | --- |
| (Intercept) | -4.35 | 0.89 | -6.08 – -2.61 | -4.90 | <0.001 |
| age centered | 0.16 | 0.24 | -0.30 – 0.62 | 0.67 | 0.504 |
| behavior code [GES_OBJ] | -0.70 | 1.04 | -2.73 – 1.33 | -0.67 | 0.501 |
| behavior code [M_ESP] | 0.91 | 0.97 | -0.99 – 2.80 | 0.94 | 0.347 |
| behavior code  [M_ESP_OBJ] | 1.38 | 1.05 | -0.67 – 3.44 | 1.32 | 0.187 |
| behavior code [M_RIT] | 0.52 | 0.90 | -1.25 – 2.29 | 0.58 | 0.564 |
| behavior code  [M_RIT_OBJ] | 1.79 | 0.90 | 0.03 – 3.55 | 1.99 | 0.046 |
| behavior code [VOC] | 1.71 | 0.89 | -0.03 – 3.46 | 1.93 | 0.054 |
| age centered × behavior  code [GES_OBJ] | 0.29 | 0.28 | -0.25 – 0.83 | 1.06 | 0.290 |
| age centered × behavior  code [M_ESP] | -0.80 | 0.26 | -1.31 – -0.28 | -3.05 | 0.002 |
| age centered × behavior  code [M_ESP_OBJ] | -0.36 | 0.30 | -0.94 – 0.22 | -1.22 | 0.224 |
| age centered × behavior  code [M_RIT] | -0.23 | 0.24 | -0.70 – 0.25 | -0.94 | 0.348 |
| age centered × behavior  code [M_RIT_OBJ] | -0.20 | 0.24 | -0.68 – 0.27 | -0.84 | 0.398 |
| age centered × behavior  code [VOC] | -0.04 | 0.24 | -0.51 – 0.42 | -0.18 | 0.860 |

**Table S2. Duration of infant behavior (Random effects)**

| σ2 | 0.63 |
| --- | --- |
| τ00 id | 0.03 |
| τ11 id.age_centered | 0.01 |
| ρ01 id | -0.41 |
| ICC | 0.13 |
| N id | 9 |
| Observations | 334 |
| Marginal R2 / Conditional R2 | 0.505 / 0.566 |
| AIC | -1156.337 |

**Table S3. Frequency of infant behavior (Fixed effects)**

| Predictors | Estimates | std. Error | CI | Statistic | p |
| --- | --- | --- | --- | --- | --- |
| (Intercept) | -0.90 | 0.75 | -2.37 – 0.58 | -1.19 | 0.235 |
| age centered | 0.08 | 0.20 | -0.32 – 0.47 | 0.38 | 0.706 |
| behavior code [GES_OBJ] | -0.71 | 0.89 | -2.45 – 1.04 | -0.80 | 0.427 |
| behavior code [M_ESP] | 0.56 | 0.82 | -1.04 – 2.16 | 0.68 | 0.496 |
| behavior code  [M_ESP_OBJ] | 0.31 | 0.86 | -1.36 – 1.99 | 0.36 | 0.715 |
| behavior code [M_RIT] | 0.16 | 0.76 | -1.33 – 1.65 | 0.21 | 0.834 |
| behavior code  [M_RIT_OBJ] | 1.20 | 0.76 | -0.29 – 2.69 | 1.58 | 0.114 |
| behavior code [VOC] | 1.78 | 0.75 | 0.30 – 3.26 | 2.36 | 0.018 |
| age centered × behavior  code [GES_OBJ] | 0.31 | 0.24 | -0.15 – 0.78 | 1.31 | 0.189 |
| age centered × behavior  code [M_ESP] | -0.30 | 0.22 | -0.73 – 0.13 | -1.37 | 0.171 |
| age centered × behavior  code [M_ESP_OBJ] | -0.08 | 0.24 | -0.54 – 0.39 | -0.33 | 0.740 |
| age centered × behavior  code [M_RIT] | -0.14 | 0.20 | -0.53 – 0.26 | -0.67 | 0.504 |
| age centered × behavior  code [M_RIT_OBJ] | -0.09 | 0.20 | -0.49 – 0.31 | -0.44 | 0.659 |
| age centered × behavior  code [VOC] | 0.03 | 0.20 | -0.36 – 0.42 | 0.13 | 0.895 |

**Table S4. Frequency of infant behavior (Random effects)**

| σ2 | 0.47 |
| --- | --- |
| τ00 id | 0.05 |
| τ11 id.age_centered | 0.00 |
| ρ01 id | -0.15 |
| ICC | 0.17 |
| N id | 9 |
| Observations | 334 |
| Marginal R2 / Conditional R2 | 0.453 / 0.543 |
| AIC | 778.178 |

**Table S5. Duration of adult behavior (Fixed effects)**

| Predictors | Estimates | std. Error | CI | Statistic | p |
| --- | --- | --- | --- | --- | --- |
| (Intercept) | -2.51 | 0.11 | -2.72 – -2.29 | -23.34 | <0.001 |
| age centered | -0.17 | 0.03 | -0.23 – -0.11 | -6.03 | <0.001 |
| behavior code [DEM_2] | -0.22 | 0.15 | -0.52 – 0.08 | -1.46 | 0.144 |
| behavior code [DEM_3] | -0.42 | 0.15 | -0.71 – -0.13 | -2.80 | 0.005 |
| behavior code [DEM_4] | -1.09 | 0.19 | -1.47 – -0.71 | -5.66 | <0.001 |
| behavior code [NRT_DIR] | -0.22 | 0.14 | -0.50 – 0.05 | -1.62 | 0.106 |
| behavior code [NRT_OBJ] | -1.54 | 0.15 | -1.83 – -1.24 | -10.14 | <0.001 |
| behavior code [RT_DIR] | -0.50 | 0.15 | -0.79 – -0.21 | -3.38 | 0.001 |
| behavior code [RT_OBJ] | -0.31 | 0.14 | -0.58 – -0.04 | -2.29 | 0.022 |
| age centered × behavior  code [DEM_2] | 0.12 | 0.04 | 0.04 – 0.21 | 2.89 | 0.004 |
| age centered × behavior  code [DEM_3] | 0.19 | 0.04 | 0.10 – 0.27 | 4.36 | <0.001 |
| age centered × behavior  code [DEM_4] | 0.11 | 0.06 | -0.02 – 0.23 | 1.67 | 0.096 |
| age centered × behavior  code [NRT_DIR] | 0.22 | 0.04 | 0.14 – 0.30 | 5.30 | <0.001 |
| age centered × behavior  code [NRT_OBJ] | -0.03 | 0.04 | -0.11 – 0.05 | -0.65 | 0.513 |
| age centered × behavior  code [RT_DIR] | 0.03 | 0.05 | -0.06 – 0.13 | 0.72 | 0.474 |
| age centered × behavior  code [RT_OBJ] | 0.05 | 0.04 | -0.04 – 0.13 | 1.08 | 0.279 |

**Table S6. Duration of adult behavior (Random effects)**

| σ2 | 0.60 |
| --- | --- |
| τ00 id | 0.02 |
| τ11 id.age_centered | 0.00 |
| ρ01 id | 1.00 |
| N id | 9 |
| Observations | 571 |
| Marginal R2 / Conditional R2 | 0.369 / NA |
| AIC | -2194.177 |

**Table S7. Frequency of adult behavior (Fixed effects)**

| Predictors | Estimates | std. Error | CI | Statistic | p |
| --- | --- | --- | --- | --- | --- |
| (Intercept) | 0.38 | 0.12 | 0.14 – 0.62 | 3.14 | 0.002 |
| age centered | -0.14 | 0.02 | -0.19 – -0.09 | -5.63 | <0.001 |
| behavior code [DEM_2] | -0.80 | 0.15 | -1.09 – -0.50 | -5.31 | <0.001 |
| behavior code [DEM_3] | -0.32 | 0.12 | -0.56 – -0.08 | -2.62 | 0.009 |
| behavior code [DEM_4] | -1.05 | 0.18 | -1.39 – -0.70 | -5.96 | <0.001 |
| behavior code [NRT_DIR] | -0.30 | 0.12 | -0.54 – -0.05 | -2.38 | 0.017 |
| behavior code [NRT_OBJ] | -1.01 | 0.13 | -1.27 – -0.75 | -7.53 | <0.001 |
| behavior code [RT_DIR] | -0.52 | 0.13 | -0.78 – -0.26 | -3.93 | <0.001 |
| behavior code [RT_OBJ] | -0.18 | 0.12 | -0.41 – 0.06 | -1.47 | 0.142 |
| age centered × behavior  code [DEM_2] | 0.29 | 0.04 | 0.20 – 0.38 | 6.54 | <0.001 |
| age centered × behavior  code [DEM_3] | 0.16 | 0.04 | 0.09 – 0.23 | 4.44 | <0.001 |
| age centered × behavior  code [DEM_4] | 0.12 | 0.06 | -0.00 – 0.23 | 1.95 | 0.051 |
| age centered × behavior  code [NRT_DIR] | 0.17 | 0.04 | 0.10 – 0.24 | 4.54 | <0.001 |
| age centered × behavior  code [NRT_OBJ] | 0.01 | 0.04 | -0.07 – 0.08 | 0.15 | 0.884 |
| age centered × behavior  code [RT_DIR] | 0.02 | 0.04 | -0.06 – 0.10 | 0.39 | 0.697 |
| age centered × behavior  code [RT_OBJ] | 0.03 | 0.04 | -0.04 – 0.11 | 0.91 | 0.362 |

**Table S8. Frequency of adult behavior (Random effects)**

| σ2 | 0.50 |
| --- | --- |
| τ00 id | 0.07 |
| τ11 id.age_centered | 0.00 |
| ρ01 id | -1.00 |
| N id | 9 |
| Observations | 583 |
| Marginal R2 / Conditional R2 | 0.297 / NA |
| AIC | 1155.537 |

**Table S9. Proportional duration of adult behaviors aggregated as distal and proximal (Fixed effects)**

| Predictors | Estimates | std. Error | CI | Statistic | p |
| --- | --- | --- | --- | --- | --- |
| (Intercept) | -2.71 | 0.07 | -2.85 – -2.57 | -38.36 | <0.001 |
| age centered | -0.08 | 0.01 | -0.11 – -0.06 | -6.53 | <0.001 |
| behaviour code [Proximal] | -0.21 | 0.08 | -0.38 – -0.05 | -2.52 | 0.012 |

**Table S10. Proportional duration of adult behaviors aggregated as distal and proximal (Random effects)**

| σ2 | 0.69 |
| --- | --- |
| τ00 id | 0.01 |
| τ11 id.age_centered | 0.01 |
| N id | 9 |
| Observations | 571 |
| ρ01 id | 0.097 / 0.109 |
| Marginal R2 / Conditional R2 | -2082.866 |
| AIC | 0.69 |

**Table S11. Frequency of adult behaviors aggregated as distal and proximal (Fixed effects)**

| Predictors | Estimates | std. Error | CI | Statistic | p |
| --- | --- | --- | --- | --- | --- |
| (Intercept) | 0.15 | 0.11 | -0.06 – 0.37 | 1.42 | 0.154 |
| age centered | -0.06 | 0.01 | -0.09 – -0.04 | -5.61 | <0.001 |
| behaviour code [Proximal] | -0.15 | 0.08 | -0.30 – -0.00 | -1.97 | 0.049 |

**Table S12. Frequency of adult behaviors aggregated as distal and proximal (Random effects)**

| σ2 | 0.59 |
| --- | --- |
| τ00 id | 0.08 |
| τ11 id.age_centered | 0.12 |
| N id | 9 |
| Observations | 583 |
| ρ01 id | 0.060 / 0.170 |
| Marginal R2 / Conditional R2 | 0.59 |
| AIC | 0.08 |

**Section S2. Longitudinal model of infant multimodal behavior**

**Table S13. Frequency of multimodal infant behavior (Fixed effects)**

| Predictors | Estimates | std. Error | CI | Statistic | p |
| --- | --- | --- | --- | --- | --- |
| (Intercept) | -1.12 | 0.30 | -1.70 – -0.54 | -3.80 | <0.001 |
| age centered | -0.37 | 0.09 | -0.54 – -0.19 | -4.11 | <0.001 |
| mov [GESTOS] | 0.96 | 0.80 | -0.60 – 2.52 | 1.20 | 0.229 |
| mov [RITM] | 0.26 | 0.32 | -0.37 – 0.88 | 0.81 | 0.419 |
| obj [SI] | -0.04 | 0.47 | -0.97 – 0.89 | -0.09 | 0.927 |
| voc [SI] | -1.01 | 0.43 | -1.85 – -0.17 | -2.36 | 0.018 |
| age centered × mov  [GESTOS] | 0.09 | 0.22 | -0.34 – 0.53 | 0.42 | 0.674 |
| age centered × mov [RITM] | 0.25 | 0.10 | 0.06 – 0.44 | 2.54 | 0.011 |
| age centered × obj [SI] | 0.29 | 0.15 | 0.00 – 0.59 | 1.97 | 0.049 |
| mov [GESTOS] × obj [SI] | -1.19 | 1.00 | -3.15 – 0.77 | -1.19 | 0.234 |
| mov [RITM] × obj [SI] | 1.04 | 0.50 | 0.05 – 2.03 | 2.06 | 0.039 |
| age centered × voc [SI] | 0.02 | 0.13 | -0.23 – 0.27 | 0.17 | 0.864 |
| mov [GESTOS] × voc [SI] | -0.04 | 1.34 | -2.67 – 2.59 | -0.03 | 0.978 |
| mov [RITM] × voc [SI] | 0.17 | 0.48 | -0.77 – 1.11 | 0.35 | 0.725 |
| obj [SI] × voc [SI] | 0.96 | 0.74 | -0.50 – 2.42 | 1.28 | 0.199 |
| (age centered × mov  [GESTOS]) × obj [SI] | 0.25 | 0.28 | -0.30 – 0.81 | 0.88 | 0.377 |
| (age centered × mov  [RITM]) × obj [SI] | -0.20 | 0.16 | -0.52 – 0.12 | -1.23 | 0.217 |
| (age centered × mov  [GESTOS]) × voc [SI] | 0.28 | 0.34 | -0.39 – 0.96 | 0.82 | 0.412 |
| (age centered × mov  [RITM]) × voc [SI] | 0.25 | 0.15 | -0.05 – 0.54 | 1.64 | 0.101 |
| (age centered × obj [SI])  × voc [SI] | 0.07 | 0.24 | -0.39 – 0.54 | 0.31 | 0.754 |
| (mov [GESTOS] × obj [SI])  × voc [SI] | -1.28 | 1.70 | -4.60 – 2.04 | -0.76 | 0.450 |
| (mov [RITM] × obj [SI]) ×  voc [SI] | -0.98 | 0.80 | -2.54 – 0.59 | -1.22 | 0.222 |
| (age centered × mov  [GESTOS] × obj [SI]) ×  voc [SI] | -0.29 | 0.45 | -1.18 – 0.60 | -0.64 | 0.522 |
| (age centered × mov  [RITM] × obj [SI]) × voc  [SI] | -0.41 | 0.26 | -0.93 – 0.10 | -1.58 | 0.115 |

**Table S14. Frequency of multimodal infant behavior (Random effects)**

| σ2 | 0.43 |
| --- | --- |
| τ00 id | 0.04 |
| τ11 id.age_centered | 0.00 |
| ρ01 id | 0.17 |
| ICC | 0.15 |
| N id | 9 |
| Observations | 374 |
| Marginal R2 / Conditional R2 | 0.442 / 0.526 |
| AIC | 238.997 |

**Section S3. Associations with language-growth trajectories**

**Table S15. Full regression models predicting production growth**

| Behavioral measure | Term | B | SE | 95% CI | *p* |
| --- | --- | --- | --- | --- | --- |
| Infant gestures without object use – proportional duration F(3,5) = 15.531, p = 0.006 R2 = 0.903, Adj. R2 = 0.845 | Intercept | -0.132 | 1.428 | [-3.803, 3.539] | 0.930 |
|  | Estimated production level at 12.3 months | 0.378 | 0.097 | [0.13, 0.626] | 0.011 |
|  | Behavioral slope | -449.066 | 818.160 | [-2552.213, 1654.082] | 0.607 |
|  | Estimated production level × Behavioral slope | 5.098 | 49.025 | [-120.924, 131.121] | 0.921 |
| Infant gestures with object use – proportional duration F(3,5) = 13.531, p = 0.008 R2 = 0.89, Adj. R2 = 0.825 | Intercept | -1.102 | 1.429 | [-4.777, 2.572] | 0.475 |
|  | Estimated production level at 12.3 months | 0.430 | 0.103 | [0.165, 0.695] | 0.009 |
|  | Behavioral slope | 107.883 | 180.319 | [-355.64, 571.407] | 0.576 |
|  | Estimated production level × Behavioral slope | -20.309 | 26.288 | [-87.884, 47.267] | 0.475 |
| Infant spontaneous movements without object use – proportional duration F(3,5) = 13.029, p = 0.008 R2 = 0.887, Adj. R2 = 0.819 | Intercept | -2.206 | 6.883 | [-19.899, 15.486] | 0.762 |
|  | Estimated production level at 12.3 months | 0.375 | 0.374 | [-0.585, 1.335] | 0.362 |
|  | Behavioral slope | -28.625 | 140.482 | [-389.745, 332.494] | 0.847 |
|  | Estimated production level × Behavioral slope | 0.202 | 7.791 | [-19.826, 20.231] | 0.980 |
| Infant spontaneous movements with object use – proportional duration F(3,5) = 38.849, p = 0.001 R2 = 0.959, Adj. R2 = 0.934 | Intercept | -0.153 | 0.846 | [-2.328, 2.023] | 0.864 |
|  | Estimated production level at 12.3 months | 0.254 | 0.058 | [0.106, 0.402] | 0.007 |
|  | Behavioral slope | 98.981 | 71.460 | [-84.712, 282.673] | 0.225 |
|  | Estimated production level × Behavioral slope | -14.590 | 5.754 | [-29.382, 0.201] | 0.052 |
| Infant rhythmic movements without object use – proportional duration F(3,5) = 28.962, p = 0.001 R2 = 0.946, Adj. R2 = 0.913 | Intercept | -0.699 | 0.709 | [-2.522, 1.124] | 0.369 |
|  | Estimated production level at 12.3 months | 0.421 | 0.055 | [0.28, 0.563] | 0.001 |
|  | Behavioral slope | -1,016.003 | 1,057.651 | [-3734.781, 1702.774] | 0.381 |
|  | Estimated production level × Behavioral slope | 12.935 | 63.047 | [-149.132, 175.002] | 0.846 |
| Infant rhythmic movements with object use – proportional duration F(3,5) = 50.96, p < .001 R2 = 0.968, Adj. R2 = 0.949 | Intercept | -0.391 | 1.170 | [-3.399, 2.617] | 0.752 |
|  | Estimated production level at 12.3 months | 0.455 | 0.058 | [0.307, 0.604] | 0.001 |
|  | Behavioral slope | 54.852 | 133.861 | [-289.249, 398.953] | 0.699 |
|  | Estimated production level × Behavioral slope | -22.554 | 8.343 | [-44.001, -1.107] | 0.043 |
| Infant vocalizations – proportional duration F(3,5) = 27.277, p = 0.002 R2 = 0.942, Adj. R2 = 0.908 | Intercept | -0.543 | 0.861 | [-2.756, 1.669] | 0.556 |
|  | Estimated production level at 12.3 months | 0.423 | 0.052 | [0.289, 0.557] | < .001 |
|  | Behavioral slope | 31.681 | 118.430 | [-272.753, 336.115] | 0.800 |
|  | Estimated production level × Behavioral slope | -10.965 | 7.474 | [-30.177, 8.246] | 0.202 |
| Infant gestures without object use – rate F(3,5) = 23.21, p = 0.002 R2 = 0.933, Adj. R2 = 0.893 | Intercept | -0.572 | 1.213 | [-3.69, 2.546] | 0.657 |
|  | Estimated production level at 12.3 months | 0.459 | 0.096 | [0.211, 0.707] | 0.005 |
|  | Behavioral slope | -4.835 | 31.904 | [-86.847, 77.177] | 0.885 |
|  | Estimated production level × Behavioral slope | -1.591 | 2.195 | [-7.233, 4.051] | 0.501 |
| Infant gestures with object use – rate F(3,5) = 16.728, p = 0.005 R2 = 0.909, Adj. R2 = 0.855 | Intercept | -1.104 | 1.326 | [-4.512, 2.304] | 0.443 |
|  | Estimated production level at 12.3 months | 0.453 | 0.090 | [0.222, 0.684] | 0.004 |
|  | Behavioral slope | 5.971 | 7.122 | [-12.336, 24.278] | 0.440 |
|  | Estimated production level × Behavioral slope | -1.245 | 0.944 | [-3.672, 1.182] | 0.245 |
| Infant spontaneous movements without object use – rate F(3,5) = 13.32, p = 0.008 R2 = 0.889, Adj. R2 = 0.822 | Intercept | -1.812 | 4.496 | [-13.37, 9.746] | 0.704 |
|  | Estimated production level at 12.3 months | 0.608 | 0.449 | [-0.546, 1.761] | 0.234 |
|  | Behavioral slope | -4.949 | 20.117 | [-56.662, 46.764] | 0.815 |
|  | Estimated production level × Behavioral slope | 1.143 | 2.092 | [-4.234, 6.52] | 0.608 |
| Infant spontaneous movements with object use – rate F(3,5) = 30.042, p = 0.001 R2 = 0.947, Adj. R2 = 0.916 | Intercept | -0.188 | 1.111 | [-3.045, 2.669] | 0.872 |
|  | Estimated production level at 12.3 months | 0.251 | 0.073 | [0.062, 0.439] | 0.019 |
|  | Behavioral slope | -5.639 | 20.940 | [-59.466, 48.188] | 0.798 |
|  | Estimated production level × Behavioral slope | -0.994 | 0.943 | [-3.419, 1.431] | 0.340 |
| Infant rhythmic movements without object use – rate F(3,5) = 19.622, p = 0.003 R2 = 0.922, Adj. R2 = 0.875 | Intercept | -0.735 | 0.871 | [-2.975, 1.505] | 0.437 |
|  | Estimated production level at 12.3 months | 0.413 | 0.076 | [0.216, 0.609] | 0.003 |
|  | Behavioral slope | -53.419 | 58.425 | [-203.604, 96.767] | 0.402 |
|  | Estimated production level × Behavioral slope | 1.250 | 3.458 | [-7.641, 10.14] | 0.733 |
| Infant rhythmic movements with object use – rate F(3,5) = 24.248, p = 0.002 R2 = 0.936, Adj. R2 = 0.897 | Intercept | -0.901 | 1.812 | [-5.558, 3.756] | 0.640 |
|  | Estimated production level at 12.3 months | 0.464 | 0.094 | [0.223, 0.704] | 0.004 |
|  | Behavioral slope | 4.244 | 11.155 | [-24.431, 32.919] | 0.719 |
|  | Estimated production level × Behavioral slope | -0.983 | 0.669 | [-2.704, 0.738] | 0.202 |
| Infant vocalizations – rate F(3,5) = 37.552, p = 0.001 R2 = 0.958, Adj. R2 = 0.932 | Intercept | -0.140 | 0.712 | [-1.971, 1.691] | 0.852 |
|  | Estimated production level at 12.3 months | 0.373 | 0.037 | [0.278, 0.468] | < .001 |
|  | Behavioral slope | 0.095 | 2.348 | [-5.941, 6.131] | 0.969 |
|  | Estimated production level × Behavioral slope | -0.220 | 0.133 | [-0.562, 0.123] | 0.160 |
| Adult Level 1 object-use demonstrations – proportional duration F(3,5) = 12.383, p = 0.009 R2 = 0.881, Adj. R2 = 0.81 | Intercept | -0.681 | 1.538 | [-4.634, 3.271] | 0.676 |
|  | Estimated production level at 12.3 months | 0.386 | 0.093 | [0.146, 0.625] | 0.009 |
|  | Behavioral slope | -4.798 | 65.652 | [-173.562, 163.966] | 0.945 |
|  | Estimated production level × Behavioral slope | 1.633 | 5.182 | [-11.688, 14.953] | 0.765 |
| Adult Level 2 object-use demonstrations – proportional duration F(3,5) = 23.567, p = 0.002 R2 = 0.934, Adj. R2 = 0.894 | Intercept | -0.771 | 0.787 | [-2.795, 1.252] | 0.372 |
|  | Estimated production level at 12.3 months | 0.370 | 0.045 | [0.253, 0.487] | < .001 |
|  | Behavioral slope | -57.958 | 75.337 | [-251.619, 135.702] | 0.476 |
|  | Estimated production level × Behavioral slope | 9.615 | 5.858 | [-5.443, 24.673] | 0.162 |
| Adult Level 3 object-use demonstrations – proportional duration F(3,5) = 22.8, p = 0.002 R2 = 0.932, Adj. R2 = 0.891 | Intercept | -0.097 | 0.913 | [-2.445, 2.25] | 0.919 |
|  | Estimated production level at 12.3 months | 0.358 | 0.047 | [0.237, 0.48] | 0.001 |
|  | Behavioral slope | 90.767 | 138.371 | [-264.927, 446.462] | 0.541 |
|  | Estimated production level × Behavioral slope | -22.388 | 13.034 | [-55.893, 11.118] | 0.147 |
| Adult Level 4 symbolic object-use demonstrations – proportional duration F(3,5) = 17.881, p = 0.004 R2 = 0.915, Adj. R2 = 0.864 | Intercept | -0.586 | 1.053 | [-3.294, 2.122] | 0.602 |
|  | Estimated production level at 12.3 months | 0.384 | 0.058 | [0.236, 0.533] | 0.001 |
|  | Behavioral slope | 58.032 | 1,115.701 | [-2809.968, 2926.032] | 0.961 |
|  | Estimated production level × Behavioral slope | -32.185 | 60.908 | [-188.754, 124.384] | 0.620 |
| Adult non-rhythmic touch without object use – proportional duration F(3,5) = 24.471, p = 0.002 R2 = 0.936, Adj. R2 = 0.898 | Intercept | 0.015 | 0.964 | [-2.463, 2.494] | 0.988 |
|  | Estimated production level at 12.3 months | 0.263 | 0.071 | [0.081, 0.446] | 0.014 |
|  | Behavioral slope | -54.954 | 189.254 | [-541.447, 431.54] | 0.783 |
|  | Estimated production level × Behavioral slope | 12.986 | 10.324 | [-13.553, 39.525] | 0.264 |
| Adult non-rhythmic touch with object use – proportional duration F(3,5) = 14.529, p = 0.007 R2 = 0.897, Adj. R2 = 0.835 | Intercept | -0.149 | 1.489 | [-3.976, 3.677] | 0.924 |
|  | Estimated production level at 12.3 months | 0.370 | 0.146 | [-0.006, 0.746] | 0.052 |
|  | Behavioral slope | 310.975 | 356.891 | [-606.441, 1228.392] | 0.423 |
|  | Estimated production level × Behavioral slope | -8.117 | 23.160 | [-67.65, 51.417] | 0.740 |
| Adult rhythmic touch without object use – proportional duration F(3,5) = 19.943, p = 0.003 R2 = 0.923, Adj. R2 = 0.877 | Intercept | 0.144 | 1.163 | [-2.847, 3.134] | 0.907 |
|  | Estimated production level at 12.3 months | 0.217 | 0.107 | [-0.058, 0.492] | 0.098 |
|  | Behavioral slope | 69.334 | 96.981 | [-179.964, 318.632] | 0.507 |
|  | Estimated production level × Behavioral slope | -21.216 | 13.466 | [-55.832, 13.401] | 0.176 |
| Adult rhythmic touch with object use – proportional duration F(3,5) = 14.149, p = 0.007 R2 = 0.895, Adj. R2 = 0.831 | Intercept | -0.167 | 1.516 | [-4.065, 3.731] | 0.917 |
|  | Estimated production level at 12.3 months | 0.294 | 0.104 | [0.026, 0.562] | 0.037 |
|  | Behavioral slope | 92.692 | 185.456 | [-384.038, 569.421] | 0.638 |
|  | Estimated production level × Behavioral slope | -10.298 | 12.885 | [-43.42, 22.825] | 0.460 |
| Adult Level 1 object-use demonstrations – rate F(3,5) = 17.61, p = 0.004 R2 = 0.914, Adj. R2 = 0.862 | Intercept | -1.003 | 1.300 | [-4.344, 2.338] | 0.475 |
|  | Estimated production level at 12.3 months | 0.454 | 0.094 | [0.214, 0.695] | 0.005 |
|  | Behavioral slope | -1.889 | 3.206 | [-10.131, 6.354] | 0.581 |
|  | Estimated production level × Behavioral slope | 0.397 | 0.323 | [-0.432, 1.227] | 0.273 |
| Adult Level 2 object-use demonstrations – rate F(3,5) = 12.386, p = 0.009 R2 = 0.881, Adj. R2 = 0.81 | Intercept | -1.205 | 1.643 | [-5.428, 3.018] | 0.496 |
|  | Estimated production level at 12.3 months | 0.380 | 0.086 | [0.158, 0.602] | 0.007 |
|  | Behavioral slope | 2.689 | 10.192 | [-23.51, 28.887] | 0.802 |
|  | Estimated production level × Behavioral slope | 0.023 | 1.014 | [-2.582, 2.628] | 0.983 |
| Adult Level 3 object-use demonstrations – rate F(3,5) = 15.711, p = 0.006 R2 = 0.904, Adj. R2 = 0.847 | Intercept | -0.910 | 0.954 | [-3.363, 1.543] | 0.384 |
|  | Estimated production level at 12.3 months | 0.325 | 0.065 | [0.159, 0.491] | 0.004 |
|  | Behavioral slope | -6.346 | 7.213 | [-24.889, 12.197] | 0.419 |
|  | Estimated production level × Behavioral slope | 1.311 | 1.124 | [-1.578, 4.2] | 0.296 |
| Adult Level 4 symbolic object-use demonstrations – rate F(3,5) = 125.699, p < .001 R2 = 0.987, Adj. R2 = 0.979 | Intercept | -0.143 | 0.538 | [-1.526, 1.24] | 0.801 |
|  | Estimated production level at 12.3 months | 0.397 | 0.026 | [0.329, 0.465] | < .001 |
|  | Behavioral slope | 9.564 | 15.590 | [-30.512, 49.64] | 0.566 |
|  | Estimated production level × Behavioral slope | -4.504 | 1.021 | [-7.128, -1.88] | 0.007 |
| Adult non-rhythmic touch without object use – rate F(3,5) = 74.836, p < .001 R2 = 0.978, Adj. R2 = 0.965 | Intercept | 0.427 | 0.524 | [-0.921, 1.775] | 0.452 |
|  | Estimated production level at 12.3 months | 0.215 | 0.042 | [0.107, 0.323] | 0.004 |
|  | Behavioral slope | 4.315 | 7.287 | [-14.417, 23.047] | 0.579 |
|  | Estimated production level × Behavioral slope | 0.887 | 0.358 | [-0.032, 1.807] | 0.056 |
| Adult non-rhythmic touch with object use – rate F(3,5) = 19.799, p = 0.003 R2 = 0.922, Adj. R2 = 0.876 | Intercept | -0.735 | 1.153 | [-3.699, 2.229] | 0.552 |
|  | Estimated production level at 12.3 months | 0.444 | 0.116 | [0.147, 0.742] | 0.012 |
|  | Behavioral slope | 12.816 | 17.929 | [-33.272, 58.904] | 0.507 |
|  | Estimated production level × Behavioral slope | 0.048 | 1.162 | [-2.938, 3.034] | 0.969 |
| Adult rhythmic touch without object use – rate F(3,5) = 19.478, p = 0.003 R2 = 0.921, Adj. R2 = 0.874 | Intercept | 0.257 | 1.168 | [-2.746, 3.26] | 0.835 |
|  | Estimated production level at 12.3 months | 0.242 | 0.095 | [-0.001, 0.485] | 0.051 |
|  | Behavioral slope | 2.024 | 4.859 | [-10.466, 14.514] | 0.694 |
|  | Estimated production level × Behavioral slope | -0.643 | 0.441 | [-1.777, 0.49] | 0.204 |
| Adult rhythmic touch with object use – rate F(3,5) = 16.442, p = 0.005 R2 = 0.908, Adj. R2 = 0.853 | Intercept | 0.580 | 1.518 | [-3.323, 4.483] | 0.718 |
|  | Estimated production level at 12.3 months | 0.210 | 0.132 | [-0.129, 0.55] | 0.172 |
|  | Behavioral slope | 6.470 | 7.189 | [-12.01, 24.95] | 0.409 |
|  | Estimated production level × Behavioral slope | -0.854 | 0.666 | [-2.566, 0.858] | 0.256 |
| Infant spontaneous movements without object use or vocalization – rate F(3,5) = 21.461, p = 0.003 R2 = 0.928, Adj. R2 = 0.885 | Intercept | -0.139 | 2.199 | [-5.791, 5.514] | 0.952 |
|  | Estimated production level at 12.3 months | 0.453 | 0.104 | [0.185, 0.72] | 0.007 |
|  | Behavioral slope | -0.898 | 11.863 | [-31.393, 29.598] | 0.943 |
|  | Estimated production level × Behavioral slope | 0.962 | 0.719 | [-0.885, 2.809] | 0.238 |
| Infant spontaneous movements with object use, without vocalization – rate F(3,5) = 27.908, p = 0.002 R2 = 0.944, Adj. R2 = 0.91 | Intercept | 0.014 | 1.098 | [-2.81, 2.837] | 0.991 |
|  | Estimated production level at 12.3 months | 0.243 | 0.076 | [0.047, 0.44] | 0.024 |
|  | Behavioral slope | 6.440 | 30.321 | [-71.503, 84.383] | 0.840 |
|  | Estimated production level × Behavioral slope | -2.120 | 1.583 | [-6.19, 1.95] | 0.238 |
| Infant spontaneous movements with object use and vocalization – rate F(3,5) = 22.571, p = 0.002 R2 = 0.931, Adj. R2 = 0.89 | Intercept | -0.116 | 1.127 | [-3.012, 2.78] | 0.922 |
|  | Estimated production level at 12.3 months | 0.270 | 0.075 | [0.077, 0.463] | 0.016 |
|  | Behavioral slope | -21.043 | 69.946 | [-200.845, 158.759] | 0.776 |
|  | Estimated production level × Behavioral slope | -1.802 | 2.649 | [-8.611, 5.007] | 0.527 |
| Infant spontaneous movements without object use, with vocalization – rate F(3,5) = 20.247, p = 0.003 R2 = 0.924, Adj. R2 = 0.878 | Intercept | 0.482 | 1.801 | [-4.148, 5.113] | 0.800 |
|  | Estimated production level at 12.3 months | 0.218 | 0.109 | [-0.061, 0.497] | 0.101 |
|  | Behavioral slope | 6.840 | 37.703 | [-90.079, 103.76] | 0.863 |
|  | Estimated production level × Behavioral slope | -1.364 | 1.438 | [-5.06, 2.332] | 0.386 |
| Infant gestures without object use or vocalization – rate F(3,5) = 38.245, p = 0.001 R2 = 0.958, Adj. R2 = 0.933 | Intercept | -1.049 | 1.029 | [-3.695, 1.598] | 0.355 |
|  | Estimated production level at 12.3 months | 0.529 | 0.083 | [0.317, 0.742] | 0.001 |
|  | Behavioral slope | 27.217 | 47.376 | [-94.568, 149.001] | 0.591 |
|  | Estimated production level × Behavioral slope | -7.566 | 3.772 | [-17.262, 2.129] | 0.101 |
| Infant gestures with object use, without vocalization – rate F(3,5) = 17.604, p = 0.004 R2 = 0.914, Adj. R2 = 0.862 | Intercept | -0.860 | 1.265 | [-4.111, 2.39] | 0.526 |
|  | Estimated production level at 12.3 months | 0.431 | 0.075 | [0.238, 0.623] | 0.002 |
|  | Behavioral slope | 6.837 | 7.654 | [-12.839, 26.512] | 0.413 |
|  | Estimated production level × Behavioral slope | -1.525 | 1.060 | [-4.249, 1.199] | 0.210 |
| Infant gestures with object use and vocalization – rate F(3,5) = 12.415, p = 0.009 R2 = 0.882, Adj. R2 = 0.811 | Intercept | -0.920 | 1.829 | [-5.621, 3.781] | 0.636 |
|  | Estimated production level at 12.3 months | 0.410 | 0.151 | [0.021, 0.798] | 0.042 |
|  | Behavioral slope | 4.221 | 60.719 | [-151.862, 160.303] | 0.947 |
|  | Estimated production level × Behavioral slope | -1.654 | 5.448 | [-15.658, 12.351] | 0.774 |
| Infant gestures without object use, with vocalization – rate F(3,5) = 25.812, p = 0.002 R2 = 0.939, Adj. R2 = 0.903 | Intercept | 0.192 | 1.011 | [-2.407, 2.79] | 0.857 |
|  | Estimated production level at 12.3 months | 0.348 | 0.077 | [0.15, 0.545] | 0.006 |
|  | Behavioral slope | -115.622 | 73.751 | [-305.205, 73.961] | 0.178 |
|  | Estimated production level × Behavioral slope | 4.483 | 4.183 | [-6.269, 15.235] | 0.333 |
| Infant rhythmic movements without object use or vocalization – rate F(3,5) = 13.116, p = 0.008 R2 = 0.887, Adj. R2 = 0.82 | Intercept | -0.647 | 1.015 | [-3.257, 1.963] | 0.552 |
|  | Estimated production level at 12.3 months | 0.344 | 0.068 | [0.17, 0.518] | 0.004 |
|  | Behavioral slope | -23.516 | 87.590 | [-248.673, 201.642] | 0.799 |
|  | Estimated production level × Behavioral slope | 2.177 | 4.928 | [-10.491, 14.846] | 0.677 |
| Infant rhythmic movements with object use, without vocalization – rate F(3,5) = 24.595, p = 0.002 R2 = 0.937, Adj. R2 = 0.898 | Intercept | -0.442 | 1.605 | [-4.567, 3.682] | 0.794 |
|  | Estimated production level at 12.3 months | 0.409 | 0.072 | [0.224, 0.595] | 0.002 |
|  | Behavioral slope | 3.009 | 10.660 | [-24.392, 30.41] | 0.789 |
|  | Estimated production level × Behavioral slope | -0.926 | 0.602 | [-2.474, 0.621] | 0.185 |
| Infant rhythmic movements with object use and vocalization – rate F(3,5) = 18.319, p = 0.004 R2 = 0.917, Adj. R2 = 0.867 | Intercept | 0.910 | 1.637 | [-3.299, 5.119] | 0.602 |
|  | Estimated production level at 12.3 months | 0.316 | 0.173 | [-0.127, 0.76] | 0.126 |
|  | Behavioral slope | -113.655 | 74.083 | [-304.09, 76.78] | 0.186 |
|  | Estimated production level × Behavioral slope | 4.074 | 4.116 | [-6.508, 14.656] | 0.368 |
| Infant rhythmic movements without object use, with vocalization – rate F(3,5) = 55.032, p < .001 R2 = 0.971, Adj. R2 = 0.953 | Intercept | -0.309 | 0.640 | [-1.955, 1.336] | 0.650 |
|  | Estimated production level at 12.3 months | 0.372 | 0.059 | [0.22, 0.523] | 0.001 |
|  | Behavioral slope | -160.942 | 116.928 | [-461.516, 139.632] | 0.227 |
|  | Estimated production level × Behavioral slope | 6.473 | 6.958 | [-11.413, 24.36] | 0.395 |
| Infant spontaneous movements – rate (aggregated across object use and vocalization) F(3,5) = 19.361, p = 0.003 R2 = 0.921, Adj. R2 = 0.873 | Intercept | 2.314 | 2.988 | [-5.367, 9.995] | 0.474 |
|  | Estimated production level at 12.3 months | -0.022 | 0.249 | [-0.662, 0.618] | 0.933 |
|  | Behavioral slope | 38.192 | 42.392 | [-70.781, 147.165] | 0.409 |
|  | Estimated production level × Behavioral slope | -5.201 | 3.323 | [-13.744, 3.342] | 0.178 |
| Infant rhythmic movements – rate (aggregated across object use and vocalization) F(3,5) = 24.89, p = 0.002 R2 = 0.937, Adj. R2 = 0.9 | Intercept | -0.665 | 1.952 | [-5.683, 4.353] | 0.747 |
|  | Estimated production level at 12.3 months | 0.463 | 0.105 | [0.194, 0.732] | 0.007 |
|  | Behavioral slope | 6.543 | 49.409 | [-120.466, 133.552] | 0.900 |
|  | Estimated production level × Behavioral slope | -2.934 | 2.787 | [-10.099, 4.23] | 0.341 |
| Infant gestures – rate (aggregated across object use and vocalization) F(3,5) = 26.029, p = 0.002 R2 = 0.94, Adj. R2 = 0.904 | Intercept | -1.243 | 1.226 | [-4.395, 1.909] | 0.357 |
|  | Estimated production level at 12.3 months | 0.519 | 0.090 | [0.287, 0.75] | 0.002 |
|  | Behavioral slope | 22.774 | 23.234 | [-36.951, 82.5] | 0.372 |
|  | Estimated production level × Behavioral slope | -5.174 | 2.466 | [-11.512, 1.165] | 0.090 |
| Infant behaviors with object use – rate F(3,5) = 71.358, p < .001 R2 = 0.977, Adj. R2 = 0.963 | Intercept | -0.165 | 0.700 | [-1.965, 1.635] | 0.823 |
|  | Estimated production level at 12.3 months | 0.406 | 0.034 | [0.318, 0.495] | < .001 |
|  | Behavioral slope | 11.767 | 11.782 | [-18.519, 42.053] | 0.364 |
|  | Estimated production level × Behavioral slope | -4.200 | 1.007 | [-6.789, -1.612] | 0.009 |
| Infant behaviors without object use – rate F(3,5) = 14.719, p = 0.006 R2 = 0.898, Adj. R2 = 0.837 | Intercept | -1.080 | 3.647 | [-10.456, 8.296] | 0.779 |
|  | Estimated production level at 12.3 months | 0.317 | 0.218 | [-0.244, 0.878] | 0.206 |
|  | Behavioral slope | -8.864 | 113.202 | [-299.858, 282.13] | 0.941 |
|  | Estimated production level × Behavioral slope | -3.006 | 7.952 | [-23.448, 17.436] | 0.721 |
| Infant behaviors with vocalization – rate F(3,5) = 52.66, p < .001 R2 = 0.969, Adj. R2 = 0.951 | Intercept | -0.302 | 0.599 | [-1.842, 1.238] | 0.635 |
|  | Estimated production level at 12.3 months | 0.356 | 0.039 | [0.256, 0.455] | < .001 |
|  | Behavioral slope | -51.800 | 102.045 | [-314.114, 210.514] | 0.633 |
|  | Estimated production level × Behavioral slope | -2.408 | 5.115 | [-15.556, 10.74] | 0.658 |
| Infant behaviors without vocalization – rate F(3,5) = 60.081, p < .001 R2 = 0.973, Adj. R2 = 0.957 | Intercept | -0.099 | 0.549 | [-1.51, 1.311] | 0.863 |
|  | Estimated production level at 12.3 months | 0.326 | 0.031 | [0.246, 0.407] | < .001 |
|  | Behavioral slope | 27.611 | 13.923 | [-8.18, 63.402] | 0.104 |
|  | Estimated production level × Behavioral slope | -7.733 | 1.890 | [-12.593, -2.874] | 0.009 |
|  | | | | | |

**Table S16. Full regression models predicting comprehension growth**

| Behavioral measure | Term | B | SE | 95% CI | *p* |
| --- | --- | --- | --- | --- | --- |
| Infant gestures without object use – proportional duration F(3,5) = 285.366, p < .001 R2 = 0.994, Adj. R2 = 0.991 | Intercept | -4.587 | 1.512 | [-8.472, -0.701] | 0.029 |
|  | Estimated comprehension level at 12.3 months | 0.323 | 0.023 | [0.265, 0.382] | < .001 |
|  | Behavioral slope | 1,118.014 | 738.529 | [-780.435, 3016.463] | 0.190 |
|  | Estimated comprehension level × Behavioral slope | -15.622 | 13.950 | [-51.482, 20.237] | 0.314 |
| Infant gestures with object use – proportional duration F(3,5) = 269.593, p < .001 R2 = 0.994, Adj. R2 = 0.99 | Intercept | -4.292 | 1.477 | [-8.088, -0.496] | 0.034 |
|  | Estimated comprehension level at 12.3 months | 0.304 | 0.013 | [0.27, 0.337] | < .001 |
|  | Behavioral slope | 140.582 | 262.454 | [-534.078, 815.241] | 0.615 |
|  | Estimated comprehension level × Behavioral slope | 1.824 | 5.781 | [-13.037, 16.686] | 0.765 |
| Infant spontaneous movements without object use – proportional duration F(3,5) = 225.53, p < .001 R2 = 0.993, Adj. R2 = 0.988 | Intercept | -5.071 | 4.729 | [-17.228, 7.086] | 0.333 |
|  | Estimated comprehension level at 12.3 months | 0.335 | 0.041 | [0.229, 0.441] | < .001 |
|  | Behavioral slope | -56.749 | 90.407 | [-289.149, 175.65] | 0.558 |
|  | Estimated comprehension level × Behavioral slope | 0.974 | 1.077 | [-1.794, 3.742] | 0.407 |
| Infant spontaneous movements with object use – proportional duration F(3,5) = 230.809, p < .001 R2 = 0.993, Adj. R2 = 0.989 | Intercept | -2.613 | 1.500 | [-6.469, 1.244] | 0.142 |
|  | Estimated comprehension level at 12.3 months | 0.305 | 0.015 | [0.267, 0.343] | < .001 |
|  | Behavioral slope | -15.201 | 189.549 | [-502.454, 472.051] | 0.939 |
|  | Estimated comprehension level × Behavioral slope | 1.042 | 2.264 | [-4.777, 6.862] | 0.665 |
| Infant rhythmic movements without object use – proportional duration F(3,5) = 337.932, p < .001 R2 = 0.995, Adj. R2 = 0.992 | Intercept | -2.720 | 1.461 | [-6.476, 1.037] | 0.122 |
|  | Estimated comprehension level at 12.3 months | 0.279 | 0.024 | [0.217, 0.341] | < .001 |
|  | Behavioral slope | 195.739 | 823.420 | [-1920.93, 2312.408] | 0.822 |
|  | Estimated comprehension level × Behavioral slope | 13.069 | 15.054 | [-25.628, 51.766] | 0.425 |
| Infant rhythmic movements with object use – proportional duration F(3,5) = 295.75, p < .001 R2 = 0.994, Adj. R2 = 0.991 | Intercept | -2.172 | 2.778 | [-9.312, 4.968] | 0.470 |
|  | Estimated comprehension level at 12.3 months | 0.256 | 0.045 | [0.141, 0.371] | 0.002 |
|  | Behavioral slope | 41.155 | 296.508 | [-721.043, 803.353] | 0.895 |
|  | Estimated comprehension level × Behavioral slope | 3.820 | 4.184 | [-6.935, 14.574] | 0.403 |
| Infant vocalizations – proportional duration F(3,5) = 240.823, p < .001 R2 = 0.993, Adj. R2 = 0.989 | Intercept | -0.504 | 2.452 | [-6.808, 5.799] | 0.845 |
|  | Estimated comprehension level at 12.3 months | 0.267 | 0.034 | [0.18, 0.355] | 0.001 |
|  | Behavioral slope | -225.986 | 218.759 | [-788.324, 336.351] | 0.349 |
|  | Estimated comprehension level × Behavioral slope | 2.493 | 2.336 | [-3.511, 8.497 | 0.335 |
| Infant gestures without object use – rate F(3,5) = 353.458, p < .001 R2 = 0.995, Adj. R2 = 0.992 | Intercept | -6.985 | 2.242 | [-12.748, -1.223] | 0.026 |
|  | Estimated comprehension level at 12.3 months | 0.387 | 0.052 | [0.253, 0.522] | 0.001 |
|  | Behavioral slope | 96.789 | 48.210 | [-27.138, 220.716] | 0.101 |
|  | Estimated comprehension level × Behavioral slope | -1.746 | 0.999 | [-4.315, 0.823] | 0.141 |
| Infant gestures with object use – rate F(3,5) = 260.63, p < .001 R2 = 0.994, Adj. R2 = 0.99 | Intercept | -4.006 | 1.498 | [-7.857, -0.155] | 0.044 |
|  | Estimated comprehension level at 12.3 months | 0.299 | 0.016 | [0.258, 0.339] | < .001 |
|  | Behavioral slope | 2.830 | 12.512 | [-29.332, 34.993] | 0.830 |
|  | Estimated comprehension level × Behavioral slope | 0.139 | 0.277 | [-0.572, 0.851] | 0.636 |
| Infant spontaneous movements without object use – rate F(3,5) = 518.49, p < .001 R2 = 0.997, Adj. R2 = 0.995 | Intercept | 9.244 | 5.053 | [-3.746, 22.235] | 0.127 |
|  | Estimated comprehension level at 12.3 months | 0.142 | 0.056 | [-0.003, 0.286] | 0.053 |
|  | Behavioral slope | 53.420 | 22.774 | [-5.122, 111.961] | 0.066 |
|  | Estimated comprehension level × Behavioral slope | -0.658 | 0.233 | [-1.256, -0.061] | 0.037 |
| Infant spontaneous movements with object use – rate F(3,5) = 211.679, p < .001 R2 = 0.992, Adj. R2 = 0.988 | Intercept | -3.296 | 1.970 | [-8.359, 1.767] | 0.155 |
|  | Estimated comprehension level at 12.3 months | 0.319 | 0.035 | [0.23, 0.408] | < .001 |
|  | Behavioral slope | -24.608 | 56.025 | [-168.624, 119.408] | 0.679 |
|  | Estimated comprehension level × Behavioral slope | 0.644 | 1.090 | [-2.159, 3.446] | 0.581 |
| Infant rhythmic movements without object use – rate F(3,5) = 286.322, p < .001 R2 = 0.994, Adj. R2 = 0.991 | Intercept | -2.542 | 1.675 | [-6.848, 1.765] | 0.190 |
|  | Estimated comprehension level at 12.3 months | 0.279 | 0.026 | [0.212, 0.347] | < .001 |
|  | Behavioral slope | 18.640 | 35.662 | [-73.031, 110.311] | 0.624 |
|  | Estimated comprehension level × Behavioral slope | 0.313 | 0.474 | [-0.906, 1.532] | 0.538 |
| Infant rhythmic movements with object use – rate F(3,5) = 301.261, p < .001 R2 = 0.994, Adj. R2 = 0.991 | Intercept | -3.158 | 2.842 | [-10.464, 4.148] | 0.317 |
|  | Estimated comprehension level at 12.3 months | 0.269 | 0.039 | [0.17, 0.369] | 0.001 |
|  | Behavioral slope | 9.476 | 16.543 | [-33.05, 52.002] | 0.592 |
|  | Estimated comprehension level × Behavioral slope | 0.096 | 0.165 | [-0.327, 0.52] | 0.584 |
| Infant vocalizations – rate F(3,5) = 253.38, p < .001 R2 = 0.993, Adj. R2 = 0.99 | Intercept | -0.632 | 2.136 | [-6.123, 4.858] | 0.779 |
|  | Estimated comprehension level at 12.3 months | 0.266 | 0.031 | [0.187, 0.345] | < .001 |
|  | Behavioral slope | -6.198 | 5.225 | [-19.63, 7.234] | 0.289 |
|  | Estimated comprehension level × Behavioral slope | 0.087 | 0.072 | [-0.099, 0.273] | 0.283 |
| Adult Level 1 object-use demonstrations – proportional duration F(3,5) = 204.336, p < .001 R2 = 0.992, Adj. R2 = 0.987 | Intercept | -3.244 | 2.686 | [-10.147, 3.66] | 0.281 |
|  | Estimated comprehension level at 12.3 months | 0.297 | 0.033 | [0.212, 0.382] | < .001 |
|  | Behavioral slope | -19.714 | 144.794 | [-391.919, 352.491] | 0.897 |
|  | Estimated comprehension level × Behavioral slope | -0.139 | 1.753 | [-4.645, 4.367] | 0.940 |
| Adult Level 2 object-use demonstrations – proportional duration F(3,5) = 194.553, p < .001 R2 = 0.992, Adj. R2 = 0.986 | Intercept | -2.815 | 1.367 | [-6.329, 0.699] | 0.095 |
|  | Estimated comprehension level at 12.3 months | 0.298 | 0.019 | [0.25, 0.346] | < .001 |
|  | Behavioral slope | -2.690 | 180.102 | [-465.656, 460.276] | 0.989 |
|  | Estimated comprehension level × Behavioral slope | 0.238 | 2.725 | [-6.767, 7.244] | 0.934 |
| Adult Level 3 object-use demonstrations – proportional duration F(3,5) = 348.988, p < .001 R2 = 0.995, Adj. R2 = 0.992 | Intercept | -3.310 | 0.992 | [-5.86, -0.761] | 0.021 |
|  | Estimated comprehension level at 12.3 months | 0.302 | 0.009 | [0.278, 0.326] | < .001 |
|  | Behavioral slope | 630.573 | 317.092 | [-184.539, 1445.685] | 0.103 |
|  | Estimated comprehension level × Behavioral slope | -6.595 | 3.739 | [-16.207, 3.016] | 0.138 |
| Adult Level 4 symbolic object-use demonstrations – proportional duration F(3,5) = 672.312, p < .001 R2 = 0.998, Adj. R2 = 0.996 | Intercept | -2.838 | 1.060 | [-5.563, -0.114] | 0.044 |
|  | Estimated comprehension level at 12.3 months | 0.276 | 0.017 | [0.234, 0.319] | < .001 |
|  | Behavioral slope | 2,564.016 | 933.104 | [165.397, 4962.635] | 0.040 |
|  | Estimated comprehension level × Behavioral slope | -4.292 | 5.518 | [-18.476, 9.893] | 0.472 |
| Adult non-rhythmic touch without object use – proportional duration F(3,5) = 280.762, p < .001 R2 = 0.994, Adj. R2 = 0.991 | Intercept | -0.775 | 1.839 | [-5.503, 3.952] | 0.691 |
|  | Estimated comprehension level at 12.3 months | 0.267 | 0.030 | [0.188, 0.345] | < .001 |
|  | Behavioral slope | -444.130 | 301.994 | [-1220.429, 332.169] | 0.201 |
|  | Estimated comprehension level × Behavioral slope | 6.640 | 5.520 | [-7.548, 20.829] | 0.283 |
| Adult non-rhythmic touch with object use – proportional duration F(3,5) = 203.779, p < .001 R2 = 0.992, Adj. R2 = 0.987 | Intercept | -4.267 | 2.932 | [-11.805, 3.27] | 0.205 |
|  | Estimated comprehension level at 12.3 months | 0.321 | 0.044 | [0.208, 0.434] | 0.001 |
|  | Behavioral slope | -282.050 | 574.097 | [-1757.813, 1193.713] | 0.644 |
|  | Estimated comprehension level × Behavioral slope | 3.883 | 7.740 | [-16.013, 23.78] | 0.637 |
| Adult rhythmic touch without object use – proportional duration F(3,5) = 277.32, p < .001 R2 = 0.994, Adj. R2 = 0.99 | Intercept | -4.106 | 1.369 | [-7.625, -0.588] | 0.030 |
|  | Estimated comprehension level at 12.3 months | 0.322 | 0.021 | [0.269, 0.375] | < .001 |
|  | Behavioral slope | -412.735 | 309.380 | [-1208.021, 382.551] | 0.240 |
|  | Estimated comprehension level × Behavioral slope | 9.228 | 8.720 | [-13.188, 31.645] | 0.338 |
| Adult rhythmic touch with object use – proportional duration F(3,5) = 207.256, p < .001 R2 = 0.992, Adj. R2 = 0.987 | Intercept | -3.379 | 2.040 | [-8.624, 1.867] | 0.159 |
|  | Estimated comprehension level at 12.3 months | 0.302 | 0.013 | [0.268, 0.336] | < .001 |
|  | Behavioral slope | -132.844 | 256.970 | [-793.407, 527.718] | 0.627 |
|  | Estimated comprehension level × Behavioral slope | 1.597 | 2.698 | [-5.339, 8.533] | 0.580 |
| Adult Level 1 object-use demonstrations – rate F(3,5) = 257.35, p < .001 R2 = 0.994, Adj. R2 = 0.99 | Intercept | -0.350 | 3.084 | [-8.279, 7.579] | 0.914 |
|  | Estimated comprehension level at 12.3 months | 0.254 | 0.043 | [0.144, 0.364] | 0.002 |
|  | Behavioral slope | 7.029 | 9.416 | [-17.175, 31.234] | 0.489 |
|  | Estimated comprehension level × Behavioral slope | -0.102 | 0.099 | [-0.357, 0.153] | 0.350 |
| Adult Level 2 object-use demonstrations – rate F(3,5) = 195.385, p < .001 R2 = 0.992, Adj. R2 = 0.986 | Intercept | -3.168 | 1.754 | [-7.675, 1.34] | 0.131 |
|  | Estimated comprehension level at 12.3 months | 0.302 | 0.024 | [0.24, 0.365] | < .001 |
|  | Behavioral slope | 3.441 | 17.512 | [-41.575, 48.457] | 0.852 |
|  | Estimated comprehension level × Behavioral slope | -0.032 | 0.259 | [-0.698, 0.635] | 0.907 |
| Adult Level 3 object-use demonstrations – rate F(3,5) = 256.513, p < .001 R2 = 0.994, Adj. R2 = 0.99 | Intercept | -3.150 | 1.106 | [-5.993, -0.307] | 0.036 |
|  | Estimated comprehension level at 12.3 months | 0.307 | 0.016 | [0.266, 0.349] | < .001 |
|  | Behavioral slope | 13.768 | 11.108 | [-14.785, 42.321] | 0.270 |
|  | Estimated comprehension level × Behavioral slope | -0.158 | 0.173 | [-0.602, 0.285] | 0.401 |
| Adult Level 4 symbolic object-use demonstrations – rate F(3,5) = 368.779, p < .001 R2 = 0.996, Adj. R2 = 0.993 | Intercept | -2.536 | 1.806 | [-7.178, 2.105] | 0.219 |
|  | Estimated comprehension level at 12.3 months | 0.271 | 0.029 | [0.197, 0.344] | < .001 |
|  | Behavioral slope | 14.207 | 54.101 | [-124.864, 153.278] | 0.803 |
|  | Estimated comprehension level × Behavioral slope | 0.709 | 0.774 | [-1.281, 2.699] | 0.402 |
| Adult non-rhythmic touch without object use – rate F(3,5) = 219.411, p < .001 R2 = 0.992, Adj. R2 = 0.988 | Intercept | -3.036 | 1.589 | [-7.12, 1.048] | 0.114 |
|  | Estimated comprehension level at 12.3 months | 0.306 | 0.017 | [0.262, 0.35] | < .001 |
|  | Behavioral slope | 18.546 | 41.390 | [-87.849, 124.942] | 0.673 |
|  | Estimated comprehension level × Behavioral slope | -0.536 | 0.866 | [-2.762, 1.689] | 0.563 |
| Adult non-rhythmic touch with object use – rate F(3,5) = 213.982, p < .001 R2 = 0.992, Adj. R2 = 0.988 | Intercept | -3.036 | 2.449 | [-9.331, 3.26] | 0.270 |
|  | Estimated comprehension level at 12.3 months | 0.292 | 0.033 | [0.207, 0.378] | < .001 |
|  | Behavioral slope | -7.221 | 20.737 | [-60.528, 46.086] | 0.742 |
|  | Estimated comprehension level × Behavioral slope | -0.015 | 0.182 | [-0.482, 0.453] | 0.939 |
| Adult rhythmic touch without object use – rate F(3,5) = 279.481, p < .001 R2 = 0.994, Adj. R2 = 0.991 | Intercept | -4.122 | 1.344 | [-7.576, -0.668] | 0.028 |
|  | Estimated comprehension level at 12.3 months | 0.305 | 0.014 | [0.268, 0.341] | < .001 |
|  | Behavioral slope | -6.180 | 18.820 | [-54.558, 42.198] | 0.756 |
|  | Estimated comprehension level × Behavioral slope | -0.058 | 0.512 | [-1.373, 1.257] | 0.914 |
| Adult rhythmic touch with object use – rate F(3,5) = 216.112, p < .001 R2 = 0.992, Adj. R2 = 0.988 | Intercept | -4.559 | 2.505 | [-10.998, 1.88] | 0.128 |
|  | Estimated comprehension level at 12.3 months | 0.309 | 0.019 | [0.261, 0.357] | < .001 |
|  | Behavioral slope | -14.868 | 21.409 | [-69.902, 40.166] | 0.518 |
|  | Estimated comprehension level × Behavioral slope | 0.120 | 0.217 | [-0.438, 0.678] | 0.605 |
| Infant spontaneous movements without object use or vocalization – rate F(3,5) = 645.307, p < .001 R2 = 0.997, Adj. R2 = 0.996 | Intercept | 5.250 | 2.542 | [-1.286, 11.785] | 0.094 |
|  | Estimated comprehension level at 12.3 months | 0.176 | 0.039 | [0.075, 0.276] | 0.007 |
|  | Behavioral slope | 43.185 | 13.760 | [7.813, 78.557] | 0.026 |
|  | Estimated comprehension level × Behavioral slope | -0.595 | 0.180 | [-1.058, -0.132] | 0.021 |
| Infant spontaneous movements with object use, without vocalization – rate F(3,5) = 225.518, p < .001 R2 = 0.993, Adj. R2 = 0.988 | Intercept | -4.314 | 2.844 | [-11.625, 2.996] | 0.190 |
|  | Estimated comprehension level at 12.3 months | 0.351 | 0.069 | [0.173, 0.529] | 0.004 |
|  | Behavioral slope | -69.689 | 115.014 | [-365.341, 225.964] | 0.571 |
|  | Estimated comprehension level × Behavioral slope | 1.833 | 2.477 | [-4.533, 8.199] | 0.492 |
| Infant spontaneous movements with object use and vocalization – rate F(3,5) = 228.565, p < .001 R2 = 0.993, Adj. R2 = 0.988 | Intercept | -3.323 | 1.435 | [-7.01, 0.365] | 0.068 |
|  | Estimated comprehension level at 12.3 months | 0.304 | 0.012 | [0.273, 0.336] | < .001 |
|  | Behavioral slope | -94.316 | 100.229 | [-351.963, 163.33] | 0.390 |
|  | Estimated comprehension level × Behavioral slope | 1.410 | 1.610 | [-2.728, 5.548] | 0.421 |
| Infant spontaneous movements without object use, with vocalization – rate F(3,5) = 276.403, p < .001 R2 = 0.994, Adj. R2 = 0.99 | Intercept | -4.469 | 2.264 | [-10.289, 1.351] | 0.105 |
|  | Estimated comprehension level at 12.3 months | 0.300 | 0.027 | [0.229, 0.371] | < .001 |
|  | Behavioral slope | -19.685 | 48.532 | [-144.442, 105.071] | 0.702 |
|  | Estimated comprehension level × Behavioral slope | -0.182 | 0.847 | [-2.36, 1.996] | 0.839 |
| Infant gestures without object use or vocalization – rate F(3,5) = 222.99, p < .001 R2 = 0.993, Adj. R2 = 0.988 | Intercept | -1.364 | 4.266 | [-12.329, 9.602] | 0.762 |
|  | Estimated comprehension level at 12.3 months | 0.238 | 0.106 | [-0.034, 0.511] | 0.074 |
|  | Behavioral slope | -17.156 | 139.173 | [-374.912, 340.6] | 0.907 |
|  | Estimated comprehension level × Behavioral slope | 1.539 | 2.918 | [-5.963, 9.04] | 0.621 |
| Infant gestures with object use, without vocalization – rate F(3,5) = 243.119, p < .001 R2 = 0.993, Adj. R2 = 0.989 | Intercept | -3.954 | 1.480 | [-7.758, -0.15] | 0.044 |
|  | Estimated comprehension level at 12.3 months | 0.302 | 0.014 | [0.267, 0.338] | < .001 |
|  | Behavioral slope | 6.248 | 13.607 | [-28.73, 41.225] | 0.665 |
|  | Estimated comprehension level × Behavioral slope | 0.060 | 0.284 | [-0.67, 0.79] | 0.841 |
| Infant gestures with object use and vocalization – rate F(3,5) = 282.452, p < .001 R2 = 0.994, Adj. R2 = 0.991 | Intercept | -3.225 | 2.342 | [-9.246, 2.796] | 0.227 |
|  | Estimated comprehension level at 12.3 months | 0.286 | 0.028 | [0.213, 0.359] | < .001 |
|  | Behavioral slope | -4.538 | 101.029 | [-264.242, 255.166] | 0.966 |
|  | Estimated comprehension level × Behavioral slope | 1.179 | 1.668 | [-3.109, 5.467] | 0.511 |
| Infant gestures without object use, with vocalization – rate F(3,5) = 322.938, p < .001 R2 = 0.995, Adj. R2 = 0.992 | Intercept | -3.950 | 1.395 | [-7.535, -0.364] | 0.037 |
|  | Estimated comprehension level at 12.3 months | 0.297 | 0.021 | [0.243, 0.351] | < .001 |
|  | Behavioral slope | 34.181 | 63.546 | [-129.168, 197.53] | 0.614 |
|  | Estimated comprehension level × Behavioral slope | 0.388 | 1.346 | [-3.072, 3.847] | 0.785 |
| Infant rhythmic movements without object use or vocalization – rate F(3,5) = 207.498, p < .001 R2 = 0.992, Adj. R2 = 0.987 | Intercept | -2.256 | 1.937 | [-7.234, 2.723] | 0.297 |
|  | Estimated comprehension level at 12.3 months | 0.286 | 0.031 | [0.206, 0.366] | < .001 |
|  | Behavioral slope | -4.732 | 58.903 | [-156.148, 146.684] | 0.939 |
|  | Estimated comprehension level × Behavioral slope | 0.385 | 0.872 | [-1.856, 2.625] | 0.677 |
| Infant rhythmic movements with object use, without vocalization – rate F(3,5) = 263.929, p < .001 R2 = 0.994, Adj. R2 = 0.99 | Intercept | -1.705 | 2.545 | [-8.247, 4.837] | 0.533 |
|  | Estimated comprehension level at 12.3 months | 0.257 | 0.042 | [0.148, 0.365] | 0.002 |
|  | Behavioral slope | 0.204 | 16.192 | [-41.418, 41.825] | 0.990 |
|  | Estimated comprehension level × Behavioral slope | 0.191 | 0.207 | [-0.34, 0.722] | 0.397 |
| Infant rhythmic movements with object use and vocalization – rate F(3,5) = 395.366, p < .001 R2 = 0.996, Adj. R2 = 0.993 | Intercept | -4.842 | 2.238 | [-10.594, 0.91] | 0.083 |
|  | Estimated comprehension level at 12.3 months | 0.298 | 0.027 | [0.229, 0.367] | < .001 |
|  | Behavioral slope | 85.529 | 67.042 | [-86.808, 257.866] | 0.258 |
|  | Estimated comprehension level × Behavioral slope | -0.042 | 0.684 | [-1.8, 1.715] | 0.953 |
| Infant rhythmic movements without object use, with vocalization – rate F(3,5) = 262.666, p < .001 R2 = 0.994, Adj. R2 = 0.99 | Intercept | -3.455 | 1.815 | [-8.12, 1.209] | 0.115 |
|  | Estimated comprehension level at 12.3 months | 0.299 | 0.024 | [0.237, 0.361] | < .001 |
|  | Behavioral slope | 32.888 | 95.896 | [-213.622, 279.397] | 0.746 |
|  | Estimated comprehension level × Behavioral slope | 0.153 | 1.523 | [-3.761, 4.068] | 0.924 |
| Infant spontaneous movements – rate (aggregated across object use and vocalization) F(3,5) = 349.842, p < .001 R2 = 0.995, Adj. R2 = 0.992 | Intercept | 5.744 | 5.017 | [-7.153, 18.642] | 0.304 |
|  | Estimated comprehension level at 12.3 months | 0.171 | 0.065 | [0.002, 0.339] | 0.048 |
|  | Behavioral slope | 131.064 | 76.063 | [-64.464, 326.591] | 0.145 |
|  | Estimated comprehension level × Behavioral slope | -1.894 | 0.949 | [-4.333, 0.545] | 0.102 |
| Infant rhythmic movements – rate (aggregated across object use and vocalization) F(3,5) = 513.433, p < .001 R2 = 0.997, Adj. R2 = 0.995 | Intercept | -5.971 | 2.467 | [-12.314, 0.372] | 0.060 |
|  | Estimated comprehension level at 12.3 months | 0.291 | 0.029 | [0.216, 0.366] | < .001 |
|  | Behavioral slope | 100.986 | 53.023 | [-35.315, 237.286] | 0.115 |
|  | Estimated comprehension level × Behavioral slope | -0.102 | 0.438 | [-1.229, 1.026] | 0.826 |
| Infant gestures – rate (aggregated across object use and vocalization) F(3,5) = 330.595, p < .001 R2 = 0.995, Adj. R2 = 0.992 | Intercept | -4.770 | 1.696 | [-9.13, -0.41] | 0.037 |
|  | Estimated comprehension level at 12.3 months | 0.299 | 0.022 | [0.241, 0.357] | < .001 |
|  | Behavioral slope | 34.006 | 45.377 | [-82.639, 150.652] | 0.487 |
|  | Estimated comprehension level × Behavioral slope | 0.239 | 0.872 | [-2.002, 2.48] | 0.795 |
| Infant behaviors with object use – rate F(3,5) = 335.084, p < .001 R2 = 0.995, Adj. R2 = 0.992 | Intercept | -1.891 | 1.916 | [-6.817, 3.035] | 0.369 |
|  | Estimated comprehension level at 12.3 months | 0.260 | 0.032 | [0.178, 0.343] | < .001 |
|  | Behavioral slope | -13.125 | 38.487 | [-112.059, 85.809] | 0.747 |
|  | Estimated comprehension level × Behavioral slope | 0.926 | 0.722 | [-0.929, 2.782] | 0.256 |
| Infant behaviors without object use – rate F(3,5) = 611.272, p < .001 R2 = 0.997, Adj. R2 = 0.996 | Intercept | 3.000 | 1.944 | [-1.996, 7.996] | 0.183 |
|  | Estimated comprehension level at 12.3 months | 0.215 | 0.030 | [0.139, 0.291] | 0.001 |
|  | Behavioral slope | 235.118 | 72.477 | [48.809, 421.427] | 0.023 |
|  | Estimated comprehension level × Behavioral slope | -3.365 | 1.135 | [-6.284, -0.447] | 0.031 |
| Infant behaviors with vocalization – rate F(3,5) = 225.13, p < .001 R2 = 0.993, Adj. R2 = 0.988 | Intercept | -2.696 | 1.590 | [-6.783, 1.391] | 0.151 |
|  | Estimated comprehension level at 12.3 months | 0.294 | 0.021 | [0.239, 0.349] | < .001 |
|  | Behavioral slope | 20.776 | 164.424 | [-401.888, 443.441] | 0.904 |
|  | Estimated comprehension level × Behavioral slope | 0.580 | 2.239 | [-5.175, 6.335] | 0.806 |
| Infant behaviors without vocalization – rate F(3,5) = 362.069, p < .001 R2 = 0.995, Adj. R2 = 0.993 | Intercept | -2.452 | 1.116 | [-5.322, 0.417] | 0.079 |
|  | Estimated comprehension level at 12.3 months | 0.283 | 0.017 | [0.241, 0.326] | < .001 |
|  | Behavioral slope | -32.785 | 60.399 | [-188.046, 122.475] | 0.611 |
|  | Estimated comprehension level × Behavioral slope | 2.028 | 1.637 | [-2.18, 6.236] | 0.270 |
|  | | | | | |

**Figure S1. Significant interactions between behavioral trajectories and estimated comprehension level at 12.3 months in models predicting comprehension growth**

*Note.* Lines represent −1 SD (red), mean (green), and +1 SD (blue) of the moderator, defined as the intercept of estimated word production level at 12.3 months (mean CDI assessment age).

Y-axis: word production slope.

X-axis: behavioral slope.

(a) Rates of infants’ spontaneous movements in the absence of object use.

(b) Rates of infants spontaneous movements in the absence of vocalizations.

(c) Infant categories aggregated on the absence of object use.
